# Towards an Omics-Guided Framework for Microbial Biological Control of Fungal and Oomycete Diseases in *Cannabis sativa*: Integrating Host and Pathogen Genomics and Microbiomes

**DOI:** 10.3390/ijms27188024

**Published:** 2026-09-09

**Authors:** Tiziana M. Sirangelo

**Affiliations:** Division Biotechnologies, ENEA-Italian National Agency for New Technologies, Energy and Sustainable Economic Development, Casaccia Research Center, Via Anguillarese 301, 00123 Rome, Italy; tiziana.sirangelo@enea.it

**Keywords:** *Cannabis sativa*, biological control, fungal and oomycete diseases, microbiome, multi-omics, host genomics, pathogen genomics, synthetic microbial communities

## Abstract

The intensification of *Cannabis sativa* cultivation has increased the need for effective and sustainable plant-health strategies. Pathogen control is particularly challenging because pesticide residues may compromise product safety and quality, while repeated chemical treatments can favour the emergence of fungicide-resistant populations. Microbial biological control agents (BCAs) represent a promising component of integrated disease management, although their discovery and validation remain largely empirical. This narrative review examines the current literature on the biological control of fungal and oomycete diseases of cannabis, with particular emphasis on *Fusarium*-associated syndromes, *Golovinomyces*-associated powdery mildew, grey mould caused by *Botrytis cinerea* and major oomycete root rots. Cannabis-specific research remains limited and heterogeneous regarding reproducible efficacy across host genotypes, pathogens, and environments; mechanisms of protection; and application-oriented assessment of biosafety, crop quality, and formulation performance. To address these gaps, available evidence is organised within a prospective multi-omics-guided framework combining host and pathogen genomic characterisation, microbiome profiling, targeted BCA isolation, strain-level genomic analysis, preliminary safety screening and comparative *in planta* evaluation. Transcriptomic, metabolomic, and microbiome analyses can complement these stages by identifying molecular and community-level patterns potentially related to direct antagonism, resource competition, host defence priming and microbiome-mediated protection. The relevance of these patterns to disease suppression requires functional validation, while the resulting evidence may inform the design and evaluation of synthetic microbial communities. Integrating omics across these stages could support more traceable and application-relevant BCA development by enabling mechanistic investigation while providing a structured roadmap for advancing biological control strategies against fungal and oomycete pathogens of cannabis.

## 1. Introduction

*Cannabis sativa* L. is an ancient multipurpose crop of Asian origin belonging to the Cannabaceae family. Its long history of domestication and selection has generated considerable genetic, morphological and chemical diversity, supporting a broad range of industrial, nutritional, medicinal and recreational uses [1]. Cannabis produces hundreds of specialised metabolites, including phytocannabinoids, terpenes, flavonoids and other phenolic compounds. More than 120 phytocannabinoids have been reported, of which Δ9-tetrahydrocannabinol (THC) and cannabidiol (CBD) are the most extensively studied and the principal targets of chemotype-oriented breeding programmes [2]. Hemp and drug-type cannabis constitute agronomic and regulatory categories within *C. sativa*, distinguished primarily by their intended use and phytocannabinoid composition. Low-THC hemp is predominantly cultivated for fibre, seeds, oil and biomass, whereas medicinal and adult-use cultivars are generally selected for the accumulation of specific phytocannabinoids in glandular trichomes of female inflorescences [1,2].

Legal restrictions historically constrained access to cannabis material and slowed advances in genetic, agronomic and plant-pathological research [3,4]. During the last two decades, however, regulatory changes in several countries and the increasing economic importance of the crop have stimulated a rapid expansion of cannabis cultivation and research [3,4]. Despite this progress, several areas of cannabis production, breeding and disease management remain relatively underexplored.

Cultivation systems range from extensive outdoor cannabis cultivation to highly controlled greenhouse and indoor production of high-cannabinoid genotypes [5,6,7]. Plant health, yield and chemical composition are shaped by interactions among genotype, developmental stage, cultivation practices, environmental conditions and plant-associated organisms [5,6]. Across these diverse production systems, the expansion and intensification of *C. sativa* cultivation have been accompanied by increased recognition of diseases affecting roots, crowns, foliage, stems and inflorescences [7]. Particularly important pathogens include *Fusarium* species and oomycetes belonging to *Pythium* [8,9]. Both groups can cause damping-off and root and crown rot, whereas *Fusarium* spp. may additionally cause vascular wilt and inflorescence diseases [8,9]. *Pythium* infections are especially important in greenhouse and hydroponic systems [9]. Representative root and crown pathogens include *Fusarium oxysporum*, members of the *F. solani* species complex, *F. proliferatum*, *Pythium myriotylum*, *P. dissotocum* and *P. aphanidermatum* [8,9]. The principal aerial diseases are powdery mildew (PM), predominantly associated with *Golovinomyces* spp., and grey mould or bud rot, caused by *Botrytis cinerea* [7,10,11]. The latter pathogen can infect leaves, stems and inflorescences and may continue to compromise product quality during harvest and postharvest handling [7,10,11]. Other pathogens, including *Sclerotinia sclerotiorum*, can cause stem and crown diseases, whereas several *Fusarium* species may also colonise floral tissues [8,12]. In addition to reducing plant growth, yield and inflorescence quality, infection by toxigenic *Fusarium* species may raise concerns about potential mycotoxin accumulation in harvested cannabis tissues [8,13].

Management of cannabis diseases relies on the integration of sanitation, pathogen-free propagation material, irrigation and humidity management, environmental control and, where authorised, chemical or reduced-risk plant-protection products [7,14]. However, disease-management options in cannabis are constrained by stringent regulatory requirements and the limited availability of registered products [7,14]. This context has encouraged the investigation of complementary microbial approaches, including bacterial and fungal treatments evaluated against *Fusarium* diseases, PM and *B. cinerea* [7,15]. The need for alternative strategies is further reinforced by concerns that repeated fungicide applications may select resistant pathogen populations and disrupt non-target microorganisms associated with plants and soil [16]. Together, these considerations support the development of disease-management strategies that are effective, compatible with product-quality standards and characterised by lower toxicological and environmental risks.

Microbial biological control agents (BCAs), including bacteria, filamentous fungi and yeasts from different plant-associated compartments, represent a promising component of integrated cannabis disease management and may also provide complementary benefits for plant growth and performance beyond pathogen suppression [17,18]. Their biocontrol activity may involve direct antagonism through antibiosis, resource competition, iron sequestration, lytic enzyme production and mycoparasitism, as well as indirect mechanisms based on the modulation and priming of host immune responses [15,17].

Cannabis-specific studies have demonstrated disease suppression by selected strains of *Bacillus* and *Pseudomonas*, as well as by fungal BCAs and commercial microbial products, against pathogens including *B. cinerea*, *F. oxysporum*, *P. myriotylum* and *Sclerotium rolfsii* [18,19,20,21]. However, this evidence is currently concentrated on a relatively narrow set of strains, pathogens, cannabis genotypes and experimental environments, providing a foundation for further investigation of the factors underlying variable efficacy. More broadly, conventional BCA discovery still relies largely on culture-dependent isolation and in vitro pathogen-inhibition assays [22,23]. Although valuable for initial screening, these approaches provide only partial information about tissue colonisation, persistence within the resident microbiome, expression of biocontrol functions *in planta* and efficacy across host genotypes and cultivation systems. Omics technologies can help address these limitations by complementing conventional screening and linking the genomic potential of candidate strains with functions expressed during plant-pathogen-BCA interactions and with changes in resident microbial communities [22,23]. Advances in high-throughput sequencing, metabolomics and microbiome analysis now allow these interacting biological components to be investigated within increasingly coordinated experimental approaches [4,22].

The growing availability of cannabis genomic and transcriptomic resources further supports such a strategy. Reference genome assemblies and complementary datasets have enabled gene annotation, variant discovery, comparative analyses and the investigation of agronomically relevant traits [24,25]. Multi-omics and genome-editing approaches have also been explored for the investigation and improvement of biomass-quality traits in energy crops, including cannabis, illustrating the broader potential of these technologies to connect genotype, molecular pathways, and agronomic phenotypes [26]. Transcriptomic and metabolomic studies have also begun to characterise tissue-specific expression, pathogen-responsive processes and the molecular basis of specialised metabolism [4,6]. Together, these resources create an opportunity for closer integration with microbiome profiling, pathogen genomics and functional BCA selection, linking studies of microbial diversity and growth-promoting phenotypes with analyses of disease suppression, mechanisms of action and product-quality outcomes [27]. However, the available evidence remains fragmented across these different levels of investigation, and a coherent framework connecting BCA discovery, mechanistic characterisation and validation is still lacking.

To develop this framework, a narrative review of the scientific literature was conducted using the Scopus and PubMed databases, supplemented where appropriate by authoritative methodological and regulatory sources. Both the formalisation and update of the search strategy and the final literature searches were completed on 26 August 2026. Searches combined terms related to *Cannabis sativa* or hemp with terms referring to fungal and oomycete diseases, biological control, plant-associated microbiomes, host and pathogen genomics, microbial genomics, multi-omics, product quality and synthetic microbial communities. The complete search strategy and Boolean expressions, together with the raw number of records retrieved from each database and the number of publications retained as relevant to each thematic search domain, are reported in Supplementary Table S1. Because individual publications could contribute to more than one thematic domain, these counts are not mutually exclusive. No lower publication-date limit was imposed. Cannabis-specific studies were prioritised, whereas studies from other plant pathosystems were included only when they provided methodological or mechanistic support for pipeline components for which cannabis-specific evidence was limited or unavailable. Reference lists, together with backward and forward citation tracking, were used to identify additional relevant publications. The selected literature was critically synthesised to distinguish cannabis-specific evidence from cross-pathosystem support and to identify methodological and knowledge gaps.

On this basis, the review identifies key bottlenecks affecting the progression of promising microbial candidates from initial discovery to reproducible *in planta* disease suppression, functional analysis, and later-stage assessment, and evaluates where omics technologies may provide complementary value. It proposes an integrated, prospective multi-omics-guided pipeline linking host and pathogen genomic characterisation, microbiome-informed candidate prioritisation and BCA discovery, comparative *in planta* evaluation, mechanistic investigation and functional testing, the future design of synthetic microbial communities (SynComs), and application-oriented validation under representative cultivation conditions. By connecting these stages within an iterative framework, the review aims to address the current fragmentation of evidence and provide a structured roadmap for progressing from largely empirical microbial screening towards more traceable and application-relevant biological control, with proposed mechanisms subjected to experimental validation.

## 2. The Cannabis Pathosystem: Major Fungal and Oomycete Diseases and Implications for Biological Control

This section focuses primarily on *Fusarium* diseases, powdery mildew (PM), and grey mould caused by *B. cinerea*, because of their phytopathological importance and the availability of cannabis-specific evidence that can inform biological control strategies. Other fungal and oomycete pathogens are discussed more briefly to provide a broader disease context.

### 2.1. Fusarium Diseases

Species belonging to the genus *Fusarium* are among the most damaging fungal pathogens affecting *C. sativa*, causing destructive diseases from propagation through flowering [7,8]. Root, crown and vascular infections are particularly important during propagation and vegetative growth and can result in substantial plant losses [7,8]. The spectrum of *Fusarium* diseases is highly heterogeneous, reflecting variation in causal species, plant developmental stage and infected tissue [8]. The recognised diversity continues to expand, as illustrated by recent reports of *F. pernambucanum* as a causal agent of bud rot and the newly described *F. flavens*, associated with stem and root rot [28,29].

Among the most frequently reported agents of root, crown and vascular disease are members of the *F. oxysporum* and *F. solani* species complexes [7,30,31], while *F. proliferatum* has been linked to crown and stem rot and pith necrosis [32]. Other species can colonise floral tissues and cause bud rot or inflorescence blight, including *F. graminearum*, which has been associated with flower blight in hemp [33]. Floral infection is particularly concerning because some cannabis-derived *Fusarium* species can produce mycotoxins [13], extending their biological significance beyond disease severity to the quality and safety of harvested material.

Marked heterogeneity is also evident within individual *Fusarium* groups. This is particularly apparent within the *F. oxysporum* species complex, which includes both pathogenic and non-pathogenic isolates. Two formae speciales, *F. oxysporum* f. sp. *cannabis* and *F. oxysporum* f. sp. *vasinfectum*, have historically been associated with cannabis wilt [8]. Although *F. oxysporum* f. sp. *cannabis* was traditionally regarded as specific to *C. sativa* [8,34], recent phylogenetic and host-range analyses indicate that cannabis-derived *F. oxysporum* isolates are genetically heterogeneous and may infect multiple plant species, questioning their collective assignment to this forma specialis [35].

Such pathogen diversity is directly relevant to biological control, particularly when considered alongside variation in host susceptibility. Differences in susceptibility to *Fusarium* diseases have been reported among cannabis genotypes, although the genetic basis of resistance remains less well characterised than for PM, for which major loci and candidate susceptibility genes have been identified [8,35,36,37,38,39]. At the pathogen level, isolates may differ in host range, pathogenic behaviour, tissue association, and secondary-metabolite production [8,13,35,40], potentially influencing their response to microbial antagonists. Consistent with this possibility, a study in a non-cannabis pathosystem reported differential inhibition of two *Fusarium* species by *Bacillus* [41]. Whether cannabis-derived *Fusarium* isolates differ in their susceptibility to individual BCAs remains insufficiently established. Taken together, variation on both sides of the host–pathogen interaction supports the evaluation of candidate BCAs across representative pathogen isolates and, where possible, multiple cannabis genotypes rather than relying on a single host–pathogen combination.

### 2.2. Powdery Mildew

Powdery mildew (PM), one of the most common foliar diseases affecting *C. sativa*, is caused by obligate biotrophic fungi of the order Erysiphales, with *Golovinomyces ambrosiae* among the principal causal agents reported in cannabis [7]. As the disease progresses, epiphytic colonies may spread from leaves to stems, floral bracts and inflorescences, compromising plant vigour and harvested-product quality [42,43,44]. Following penetration of epidermal cells, the pathogen establishes haustoria that acquire nutrients from living host cells [45]. This dependence on living host tissue distinguishes PM from many necrotrophic and soil-borne cannabis pathogens and makes host physiology and defence responses important components of disease development and biological control.

In hop, PM infection has been associated with altered expression of defence-related genes encoding a chitinase and a thaumatin-like protein [46], suggesting that host transcriptional responses may also represent relevant processes to investigate in the *Cannabis-Golovinomyces* interaction. These defence responses are directly pertinent to biological control, because microbial protection may result not only from direct interference with pathogen establishment but also from modulation or priming of host defences. The contribution of these host-mediated mechanisms may vary among cannabis genotypes, which differ in susceptibility to PM and for which both major resistance loci and candidate susceptibility genes have been identified, as discussed in Section 4.1 [36,37,38,39]. This genetic variation raises the question of whether differences in host resistance are accompanied by corresponding variation in BCA-mediated protection.

This host dependence also has direct methodological implications for BCA evaluation. Because PM fungi require living plant tissue to complete their infection cycle [45], conventional in vitro antagonism assays provide only limited information on interactions involving host responses, tissue colonisation or competition at the plant surface. Initial screening should therefore be followed by host-based assays, including whole-plant systems [44], to evaluate BCA performance under biologically relevant conditions. When combined with appropriate strain-tracking methods, such experiments can assess disease progression and BCA establishment concurrently and may reveal protective effects not captured by in vitro antagonism alone. Where feasible, incorporating cannabis genotypes with contrasting levels of PM susceptibility may further help determine whether protective efficacy varies with host genetic background [36,37,38,39].

### 2.3. Botrytis Cinerea

*Botrytis cinerea* is a broad-host-range necrotrophic fungus responsible for grey mould or bud rot in *C. sativa*. It primarily affects leaves, stems and inflorescences, and can also cause seedling damping-off and leaf blight [7,11]. It is particularly damaging during flowering, when dense inflorescences provide favourable conditions for pathogen colonisation [7,10,11,43]. Initial symptoms generally include water-soaked or brown lesions that progressively develop into extensive tissue necrosis, soft decay and abundant grey conidial sporulation, substantially reducing yield, visual quality and the commercial value of harvested material [10,11].

Airborne conidia generally initiate infection by germinating on susceptible plant surfaces and penetrating directly through the cuticle, natural openings or wounded and senescing tissues [47,48]. In cannabis, disease development is favoured by high relative humidity and moderate temperatures, while the compact architecture of inflorescences may create a humid microenvironment that promotes conidial germination, tissue colonisation and sporulation [11]. This microenvironment should therefore be represented explicitly during the evaluation of grey mould control strategies.

Successful infection relies on a coordinated repertoire of virulence factors, including plant cell-wall-degrading enzymes, secreted proteins, phytotoxic secondary metabolites and processes involving the production, tolerance and manipulation of reactive oxygen species [47,48]. The relative contribution of individual virulence factors may differ among fungal isolates and according to the infected host and tissue [47,48], introducing an additional source of biological variation that may influence both disease development and BCA efficacy. However, cannabis-specific studies directly comparing BCA performance across multiple *B. cinerea* isolates remain limited.

Together, the infection biology of *B. cinerea*, the environmental conditions favouring disease and the biological variation among fungal isolates have direct implications for BCA selection and evaluation. Candidate BCAs intended for direct suppression of grey mould may need to establish and remain active on leaves and inflorescences, compete for nutrients and infection sites, and express antifungal functions within the aerial-tissue environment [18,19]. Protection may also involve modulation or priming of host defence responses [15]. Accordingly, foliar assays can be complemented by spatially separated treatments, such as root application of BCAs followed by foliar *B. cinerea* challenge, to investigate the contribution of systemic host-mediated protection independently of direct BCA-pathogen contact [15].

### 2.4. Other Fungal and Oomycete Diseases

In addition to *Fusarium* diseases, PM and grey mould, cannabis production can be affected by several other fungal and oomycete pathogens, including *Pythium* spp. and *Rhizoctonia solani*, which are primarily associated with root and crown diseases [9,49]. Pathogenicity studies have shown that multiple *Pythium* species can induce root browning and decay, stunting, wilting and plant mortality in greenhouse-grown cannabis, with root injury and prolonged substrate saturation further favouring disease development [9]. *Rhizoctonia solani* has likewise been associated with root rot and lower-stem lesions, including sore shin symptoms [49].

Other pathogens primarily affect crowns, stems and aerial tissues, including *Sclerotinia minor*, which has been reported to cause crown rot and plant collapse in hemp, and *S. sclerotiorum*, which is associated with stem canker and progressive tissue collapse [12,50]. *Neofusicoccum parvum* represents another stem pathogen and has been identified as a causal agent of stem canker and terminal dieback in cannabis [51]. The availability of genome sequences from four cannabis-derived pathogenic *N. parvum* isolates further provides a basis for investigating pathogen diversity and virulence-associated traits, while also enabling future comparisons of isolate sensitivity to candidate BCAs [51].

The diversity of infection sites and ecological requirements across these pathogens has direct implications for biological control. Root-colonising BCAs and seed, cutting or substrate treatments may be particularly relevant for soil-borne pathogens such as *Pythium* and *Rhizoctonia*, whereas pathogens affecting stems and other aerial tissues require microbial antagonists capable of reaching, establishing and remaining active at the corresponding infection sites. BCA selection and delivery strategies can therefore be tailored to the target pathogen, affected plant compartment and environmental conditions favouring disease development. Taken together, the host, pathogen and environmental heterogeneity described above indicates that BCA efficacy observed in a single experimental combination cannot be assumed to generalise across cannabis pathosystems, providing a rationale for evaluation across representative host genotypes, pathogen isolates and cultivation systems.

The pathogen-specific considerations discussed in this section are summarised in Table 1.

## 3. Current Biological Control Strategies in Cannabis

Microbial biological control in cannabis can be broadly organised into two principal strategies: direct pathogen antagonism and host-mediated protection. Plant growth promotion and stress mitigation may provide complementary benefits by supporting plant vigour and resilience, but are best considered separately from direct evidence of disease suppression.

### 3.1. Direct Antagonism of Plant Pathogens

Direct biological control occurs when a microorganism limits pathogen growth or colonisation through competition for resources and infection sites or through the production of inhibitory compounds [54,55,56]. In densely populated plant environments such as the rhizosphere and phyllosphere, occupation of potential infection sites and rapid depletion of limiting nutrients can restrict pathogen establishment [55]. Rapidly growing beneficial microorganisms may also form persistent biofilms or consume nutrient sources that would otherwise support pathogen germination and colonisation [55,57]. Microbial antagonists can also produce a wide range of diffusible and volatile antimicrobial compounds. These include phenazines, polyketides, bacteriocins, cyclic lipopeptides, other non-ribosomal peptides and volatile organic compounds [57,58,59]. Lipopeptides such as surfactins, iturins and fengycins are frequently associated with *Bacillus*-mediated suppression of fungal pathogens [60,61]. *Pseudomonas* strains can produce several antifungal metabolites, including phenazines, pyrrolnitrin, cyclic lipopeptides, pyoluteorin and 2,4-diacetylphloroglucinol [56]. In cannabis, pyoluteorin and 2,4-diacetylphloroglucinol were identified as major contributors to the suppression of *B. cinerea* by *Pseudomonas protegens* Pf-5 [19].

Beyond antimicrobial-compound production, fungal BCAs may directly attack pathogens by secreting hydrolytic enzymes, including chitinases, β-1,3-glucanases and proteases, and by engaging in mycoparasitism through attachment, coiling, penetration and degradation of pathogen structures, as documented particularly for *Trichoderma* spp. [62,63,64,65].

The relative contribution and effectiveness of these mechanisms depend on both the target pathogen and the plant compartment in which the interaction occurs. Efficient colonisation and biofilm formation may be especially important for root- and substrate-colonising BCAs [66,67], whereas BCAs targeting foliar or inflorescence pathogens need to establish, persist and express antagonistic functions on aerial tissues under the prevailing environmental conditions.

Cannabis-specific evidence for direct antagonistic activity on aerial tissues includes leaf-based and detached-inflorescence assays [10,18,19]. However, the contribution of specific antagonistic traits to disease suppression has been functionally demonstrated in only a limited number of cannabis BCA-pathogen systems [19].

### 3.2. Host-Mediated Protection and Defence Priming

In addition to suppressing pathogens through direct antagonism, beneficial microorganisms may protect *C. sativa* by modulating or priming host defences [15,54,55,56]. This host-mediated protection may be particularly relevant when the BCA and the pathogen occupy different plant compartments, for example when root-colonising microorganisms influence resistance to pathogens infecting leaves or inflorescences.

Such protection depends on the recognition of microbial or damage-derived signals and their translation into downstream defence responses [68,69,70,71]. Cell-surface pattern-recognition receptors (PRRs) recognise pathogen-associated molecular patterns (PAMPs) and damage-associated molecular patterns, whereas intracellular nucleotide-binding leucine-rich repeat receptors (NLRs) recognise pathogen effectors or effector-induced perturbations [68,69,70,71]. These interconnected immune layers trigger early signalling events, including ion fluxes, increases in cytosolic Ca^2+^, production of reactive oxygen and nitrogen species and activation of CDPK and MAPK cascades [68,72,73,74,75]. Downstream responses include transcriptional and metabolic reprogramming, accumulation of defence-related proteins and specialised metabolites, and reinforcement of structural barriers through callose deposition and lignification [76,77]. Beneficial microorganisms can induce systemic resistance (ISR) or establish a primed state in which defence responses are activated more rapidly or strongly following pathogen challenge [54,55,56,78,79]. Classical induced systemic resistance triggered by rhizobacteria such as *Pseudomonas fluorescens* WCS417 is predominantly related to jasmonic acid (JA) and ethylene (ET) signalling and requires NPR1 [78,79], whereas systemic acquired resistance (SAR) is more closely linked to salicylic acid (SA), N-hydroxypipecolic acid (NHP) and NPR1 [80,81,82]. However, these pathways are not completely independent, and beneficial microorganisms may activate or prime overlapping hormone-dependent responses [78,79,80,81,82,83].

Defence priming and ISR therefore represent related but distinct features of BCA-mediated protection. Priming is characterised by limited or transient activation following BCA treatment alone and an earlier, stronger or more sustained response after pathogen challenge compared with pathogen-only plants, whereas ISR denotes enhanced resistance in tissues spatially separated from the site of beneficial-microbe application [54,55,56,78,79]. Sustained defence activation before pathogen exposure would instead be more consistent with direct induction. Priming may thus contribute to ISR without being synonymous with it, and its protective outcome is likely to depend on pathogen lifestyle [76,84,85]. Rapid and spatially restricted hypersensitive cell death, papilla formation and early callose deposition can restrict obligate biotrophs such as PM fungi [76,84], whereas extensive host–cell death may favour necrotrophic pathogens such as *B. cinerea* [85]. For *Fusarium* diseases, interpretation may be further complicated by variation among cannabis-associated species and isolates in tissue tropism, host range and pathogenic characteristics [8,35,40], providing a rationale for testing whether host-mediated protection is consistent across different challenge isolates.

These distinctions also have implications for experimental design. Immune-related responses are most informative when interpreted together with pathogen biomass, disease progression and BCA colonisation. Untreated, BCA-only, pathogen-only and combined BCA-pathogen treatments should therefore be sampled at multiple time points before and after pathogen challenge to distinguish constitutive or directly induced defence activation from priming and pathogen responses. When systemic protection is assessed, spatial separation of the BCA and pathogen can help distinguish host-mediated protection from direct antagonism.

Cannabis-specific investigation of host-mediated protection has included targeted analyses of defence responses following root application of bacterial BCAs and subsequent foliar pathogen challenge [15], while studies of bacterial bioprotection, induced resistance and beneficial *Pseudomonas*-plant interactions provide a broader mechanistic framework [54,55,56]. Pathogenesis-related proteins PR1 and PR2, ethylene response factor 1 (ERF1), hevein-like protein (HEL), and phenylalanine ammonia-lyase (PAL) provide currently available candidate defence markers, as their expression has been characterised during *B. cinerea* infection in cannabis [15]. In particular, PR1 and PR2 can provide information on SA responses, whereas ERF1 and HEL can inform JA/ET signalling, with PAL providing a broader readout of phenylpropanoid- and defence-related metabolism [15,78,79,80,81,82]. Considered together, these markers can provide a broader view of defence pathway activation than any single transcript alone. However, no individual marker should be considered diagnostic of ISR or priming. Their interpretation should instead rely on temporal response patterns and be related to pathogen biomass and disease progression: elevated pre-challenge expression would be more consistent with direct defence induction, whereas limited pre-challenge activation followed by earlier, stronger or more sustained post-challenge responses would support a primed state.

Overall, cannabis-specific evidence directly resolving the mechanisms underlying BCA-mediated disease suppression remains limited. Among the studies considered here, experimental investigation has principally involved targeted analysis of host defence responses [15] and microbial genetic perturbation to assess the contribution of specific antimicrobial metabolites [19]. These complementary approaches have so far been applied to only a small number of BCA-pathogen combinations. Molecular associations, including changes in defence-marker expression, do not by themselves establish a causal contribution to disease suppression, which requires confirmation through functional experiments.

### 3.3. Plant Growth Promotion and Stress Mitigation

Plant growth-promoting microorganisms (PGPMs), including beneficial plant-associated bacteria, can enhance plant development through biological nitrogen fixation, phosphate solubilisation, siderophore-mediated iron acquisition, production or modulation of auxins and other phytohormones, modification of root-system architecture and degradation of 1-aminocyclopropane-1-carboxylate by ACC deaminase [86,87,88,89,90].

In *C. sativa*, inoculation with selected plant growth-promoting rhizobacteria (PGPR) consortia or individual strains has promoted root colonisation, root development, biomass accumulation, physiological performance and flower yield, although responses varied according to the microbial strain and cultivation conditions [91,92]. More broadly, some PGPMs can enhance tolerance to abiotic stresses such as salinity and drought [93].

Although these growth-promoting and stress-mitigating effects could mitigate the impact of disease or support recovery following infection, they are best regarded as complementary outcomes rather than as proxies for biological control. Plant growth promotion, pathogen suppression, host colonisation and immune modulation can therefore be assessed as distinct, although potentially interacting, outcomes. Some microbial traits may contribute simultaneously to more than one functional category. For example, siderophore production may restrict pathogens through competition for iron [94], while microbial siderophores can also improve plant iron nutrition when the host is able to access the chelated iron [90]. Similarly, microbially produced auxin can modify root architecture and facilitate the establishment of beneficial bacteria on plant roots [87,95]. Whether such effects materially contribute to disease tolerance or interact with direct and host-mediated biocontrol mechanisms remains insufficiently characterised in cannabis.

**Figure 1:**
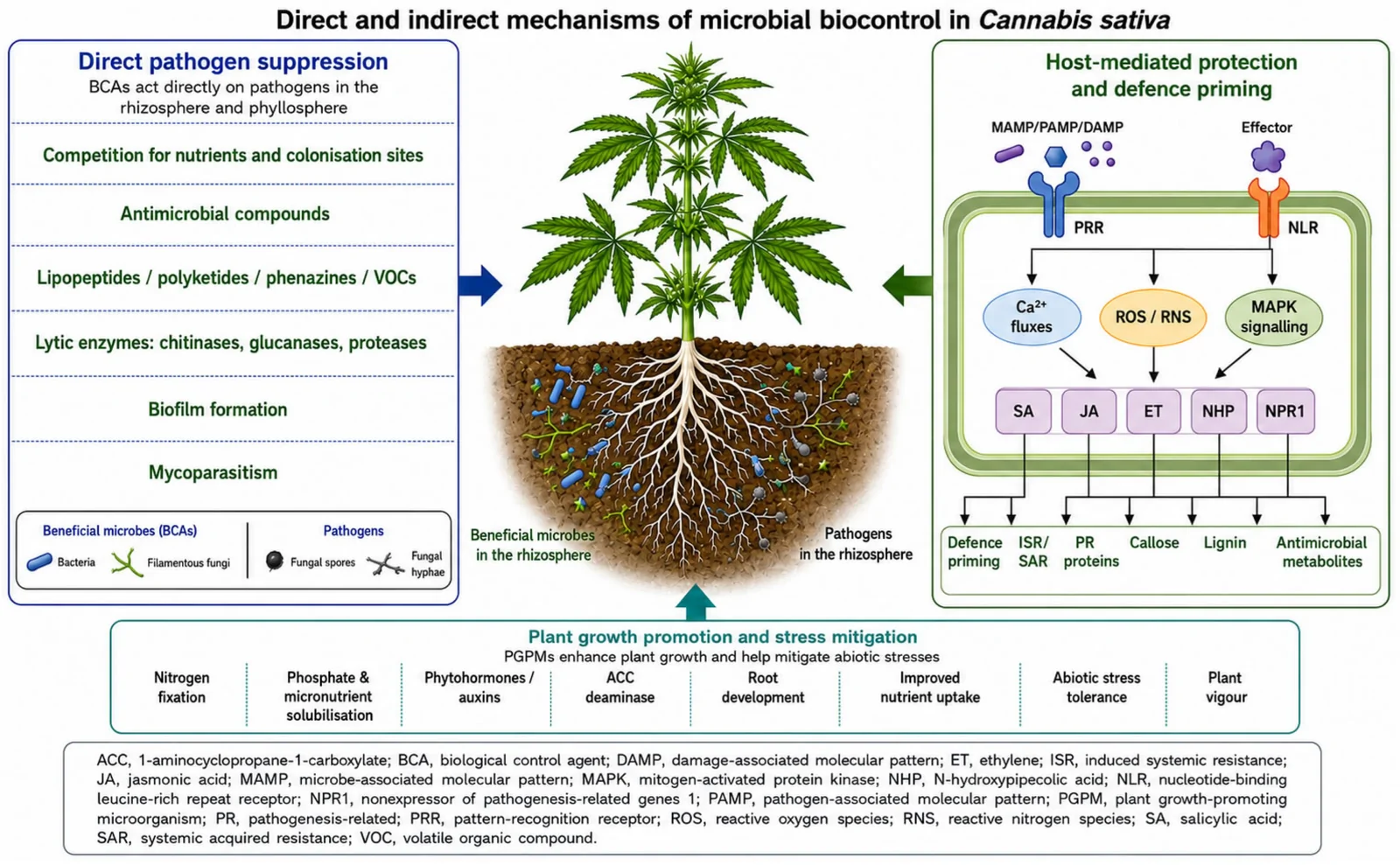
Direct and indirect mechanisms of microbial biocontrol in *Cannabis sativa*.

Figure 1 summarises the two major biocontrol strategies discussed above, direct pathogen antagonism and host-mediated protection, together with the complementary contributions of plant growth promotion and stress mitigation to plant health and performance in cannabis.

### 3.4. Evidence for Microbial Biocontrol in Cannabis sativa

The evidence for microbial biocontrol in *C. sativa* is expanding, although cannabis-specific studies remain relatively limited [96,97]. Recent reviews have summarised microbial agents proposed for the control of major cannabis pathogens and the beneficial roles of cannabis-associated endophytes, while highlighting opportunities for further mechanistic investigation and validation under cannabis cultivation conditions [96,97]. Much of the available evidence derives from in vitro screening and controlled *in planta* assays conducted in growth chambers, indoor cultivation facilities or greenhouses, whereas BCA establishment, persistence and disease suppression have been evaluated much less extensively under application-relevant cultivation and open-field conditions [96,97].

Cannabis-specific studies have explored a range of microbial interventions against soil- and seedling-associated diseases. A bacterial consortium composed of *Burkholderia ambifaria*, *Gluconacetobacter diazotrophicus* and *Herbaspirillum seropedicae* showed substantial inhibitory activity against an isolate designated by the authors as *F. oxysporum* f. sp. *cannabis* and reduced fungal infection in pre-emergence and post-emergence assays [98]. Seed-borne microorganisms represent a further source of potential antagonists. Dumigan and Deyholos [99] identified bioactive bacterial endophytes, predominantly belonging to *Bacillus* and *Paenibacillus*, that were transmitted to emerging cannabis seedlings. Several isolates inhibited seed-associated fungi, including *Fusarium* spp., suggesting potential for further evaluation in early-stage disease protection and seed-based applications.

Commercial microbial formulations have also provided controlled *in planta* evidence against soil-borne diseases. Scott and Punja [20] evaluated five products containing *B. subtilis*, *B. amyloliquefaciens*, *Gliocladium catenulatum*, *T. harzianum*, *T. virens,* or *T. asperellum* against *F. oxysporum*-induced damping-off and *P. myriotylum*-induced root and crown rot. Lalstop, RootShield, Asperello and Stargus significantly reduced disease severity caused by *F. oxysporum*, while RootShield and Lalstop were the most effective treatments overall against *P. myriotylum*. Preventive application before pathogen inoculation, together with the recovery of several of these microorganisms from internal plant tissues, suggested a potential contribution of pre-emptive colonisation to disease suppression [20].

For PM, cannabis-specific evidence derives mainly from evaluations of commercial microbial products under indoor or greenhouse conditions. Scott and Punja [44] found that repeated foliar applications of Rhapsody ASO, containing *B. subtilis* QST 713, and Stargus, containing *B. amyloliquefaciens* F727, significantly reduced PM development on the susceptible cultivar ‘Copenhagen Kush’, whereas Actinovate, based on *Streptomyces lydicus* WYEC 108, produced smaller and more variable reductions under the tested conditions. The same study also identified substantial differences in disease severity among cannabis genotypes [44]. Consistent with these findings, greenhouse experiments in hemp showed that several commercial organic fungicides, including two formulations based on *B. amyloliquefaciens*, significantly reduced PM caused by *G. ambrosiae* [52].

Microbial antagonists have likewise shown activity against pathogens affecting cannabis leaves and inflorescences. Several *Bacillus* and *Pseudomonas* strains inhibited *B. cinerea* and other cannabis fungal pathogens in vitro, and five of the six strains advanced to *in planta* testing and significantly reduced grey mould severity on cannabis leaves [18]. *Trichoderma asperellum* inhibited the in vitro growth of both *B. cinerea* and *S. sclerotiorum* and, when applied to detached cannabis inflorescences 48 h before *B. cinerea* inoculation, significantly reduced disease development [10]. However, prolific growth and sporulation of the antagonist were also observed on treated bud tissues [10]. Taken together, these studies provide evidence that both bacterial and fungal antagonists can suppress grey mould at different aerial infection sites [10,18], while highlighting the need to assess residual microbial load when BCAs are applied to inflorescences.

Studies of *Trichoderma-cannabis* interactions have also identified effects on plant performance, including plant growth, inflorescence traits and CBD content [100]. Although disease suppression was not assessed, these findings support the inclusion of crop performance and chemistry as complementary endpoints in BCA evaluation.

More broadly, in vitro antagonism should be regarded primarily as a preliminary screening and prioritisation tool rather than as evidence of biocontrol efficacy. Such assays simplify the BCA-pathogen interaction and do not reproduce several determinants of performance *in planta*, including host-mediated responses, the ability of the BCA to colonise and persist at the target tissue, interactions with the resident microbiome and the physicochemical conditions prevailing at the infection site [22,23]. Consequently, strong pathogen inhibition in vitro may not translate into disease suppression *in planta*, whereas candidates showing limited direct antagonism may still provide protection through host-mediated or other context-dependent mechanisms [54,55,56]. Advancement of candidate BCAs should therefore depend on reproducible disease suppression in host-based assays rather than on in vitro inhibition alone.

Taken together, cannabis-specific studies provide evidence ranging from in vitro antagonistic activity to disease suppression in controlled *in planta* and greenhouse assays. Across these studies, efficacy varied according to microbial strain, target pathogen, host genotype, application strategy and experimental conditions [18,20,44,52,98], while reproducible performance across broader biological and environmental diversity remains insufficiently established. Mechanistic evidence is available for only a few BCA-pathogen interactions [15,19], while the functional basis of protection remains poorly resolved for most candidates. Application-oriented assessment remains incomplete: disease suppression on cannabis inflorescences may be accompanied by substantial BCA growth and sporulation [10], whereas microbial inoculation can also affect crop-related traits, including cannabinoid content, even when disease suppression is not evaluated [100]. Cannabis-specific efficacy studies have likewise provided relatively little information on BCA biosafety and formulation-related performance. Collectively, these findings provide an empirical foundation for the prospective, more systematic, omics-guided discovery and validation framework developed in Section 4.

Table 2 summarises the cannabis-specific evidence discussed above, comparing the microbial agents evaluated, target pathogens, experimental systems, application strategies, main reported outcomes and experimental evidence. The table also explicitly distinguishes in vitro antagonism, detached-tissue assays, controlled whole-plant experiments, greenhouse evaluations, and mechanistically supported *in planta* findings. These systems should not be interpreted as providing equivalent evidentiary support: in vitro antagonism represents preliminary screening evidence, whereas replicated disease suppression in the host plant provides stronger efficacy evidence.

## 4. Omics-Guided Discovery and Development of Biological Control Agents for *Cannabis sativa*

Much of the cannabis-specific evidence derives from the evaluation of culturable microbial strains or commercial microbial products using in vitro screening and controlled *in planta* assays [10,18,19,20,52,98]. These approaches have identified bacterial and fungal agents capable of suppressing important cannabis pathogens, including *F. oxysporum*, *B. cinerea* and *G. ambrosiae.* Although they provide valuable initial evidence, such experiments alone do not establish whether promising BCAs remain active at target tissues or perform reproducibly across broader biological and environmental variation.

The studies reviewed above also reveal three interconnected bottlenecks. First, reproducible efficacy across host genotypes, pathogen isolates and cultivation environments remains insufficiently established [18,20,44,52,98]. Second, mechanistic evidence is available for only a small number of BCA-pathogen combinations [15,19], leaving the causal basis of protection unresolved for most candidates. Third, application-oriented evidence remains fragmented, with existing studies highlighting potential concerns related to microbiological quality [10] and crop-chemistry compatibility [100], whereas biosafety and formulation performance have received comparatively limited cannabis-specific evaluation. Together, these limitations constrain the progression of promising microbial candidates from initial screening to robust and application-relevant biological control.

Conventional and omics-guided approaches offer complementary advantages and limitations in BCA discovery and development. Culture-dependent isolation and in vitro antagonism assays are comparatively simple, inexpensive, and amenable to high-throughput screening, making them well suited to the initial identification and prioritisation of candidate strains [22,23]. However, they provide only partial information on traits expressed *in planta*, host- and microbiome-mediated effects, strain persistence and the molecular processes associated with disease suppression. Omics-based methods can provide additional resolution by linking genomic potential, functional activity, host responses and community-level changes [23], while also supporting strain traceability, experimental stratification, mechanistic hypothesis generation and preliminary hazard identification. Their implementation, however, requires greater analytical infrastructure, bioinformatic expertise and experimental validation and is generally associated with higher upfront costs and complexity. Their value is therefore context-dependent, and cannabis-specific evidence does not currently demonstrate that omics-guided workflows reduce development time or cost or outperform conventional screening. Omics should thus be incorporated into BCA development rather than viewed as a replacement for conventional microbiological, pathological and *in planta* validation approaches. Accordingly, omics-derived features should not be interpreted as linearly predictive of BCA efficacy, which remains an emergent and context-dependent property requiring experimental validation under biologically and environmentally relevant conditions.

Against this background, general methodological studies of beneficial plant-microbe interactions have highlighted the value of combining omics and imaging approaches to connect microbial functional traits with plant responses [23]. Building on cannabis-specific evidence together with methodological support from other plant pathosystems, this review proposes a staged omics-guided pipeline tailored to cannabis biological control, combining microbiome profiling, targeted microbial isolation, BCA and pathogen genomics, transcriptomics, proteomics, metabolomics and microscopy-based analyses with conventional plant pathology experiments. The overall objective is to integrate host, microbiome, BCA and pathogen information within a staged framework that progresses from candidate discovery and prioritisation to comparative *in planta* evaluation and later-stage validation.

The framework is organised as a stepwise process rather than as a collection of independent analytical tools. It begins with host genomic characterisation and the selection of genetically informative cannabis material, followed by controlled comparisons of healthy and diseased plants sampled from compartments affected by the target pathogen. Microbiome profiling can then identify taxa and community structures linked to plant health and, where supported by the analytical approach, suggest functional features potentially associated with disease suppression. These findings can support the prioritisation of taxa for targeted cultivation and recovery of potential BCAs, which are subsequently characterised taxonomically, phenotypically and genomically for traits related to biocontrol and adaptation to the target plant compartment, while also undergoing preliminary safety screening. Pathogen genomic and taxonomic analysis can then support the selection of representative isolates for comparative *in planta* testing. At this first validation stage, disease suppression should be assessed together with crop-chemistry compatibility, particularly cannabinoid and terpene profiles, so that candidates causing undesirable alterations in the target chemotype can be excluded before mechanistic investigation or later application-oriented validation. Once promising BCA-pathogen combinations have been selected, multi-omics datasets can be used to identify and prioritise mechanistic hypotheses involving microbial activity and host responses. When coupled with appropriately designed compartmentalised and functional experiments, these hypotheses can then be tested to assess the relative contributions of direct antagonism and host-mediated protection. The resulting evidence can inform the selection of individual BCAs or, prospectively, the rational design and experimental testing of synthetic microbial communities. Promising strains or consortia can subsequently progress to formulation development and application-oriented validation under cultivation conditions representative of their intended use and, where relevant, in the field.

Importantly, this framework should be interpreted as a prospective research roadmap rather than as an established cannabis-specific discovery-to-application workflow, because the complete sequence has not yet been experimentally validated in cannabis.

**Figure 2:**
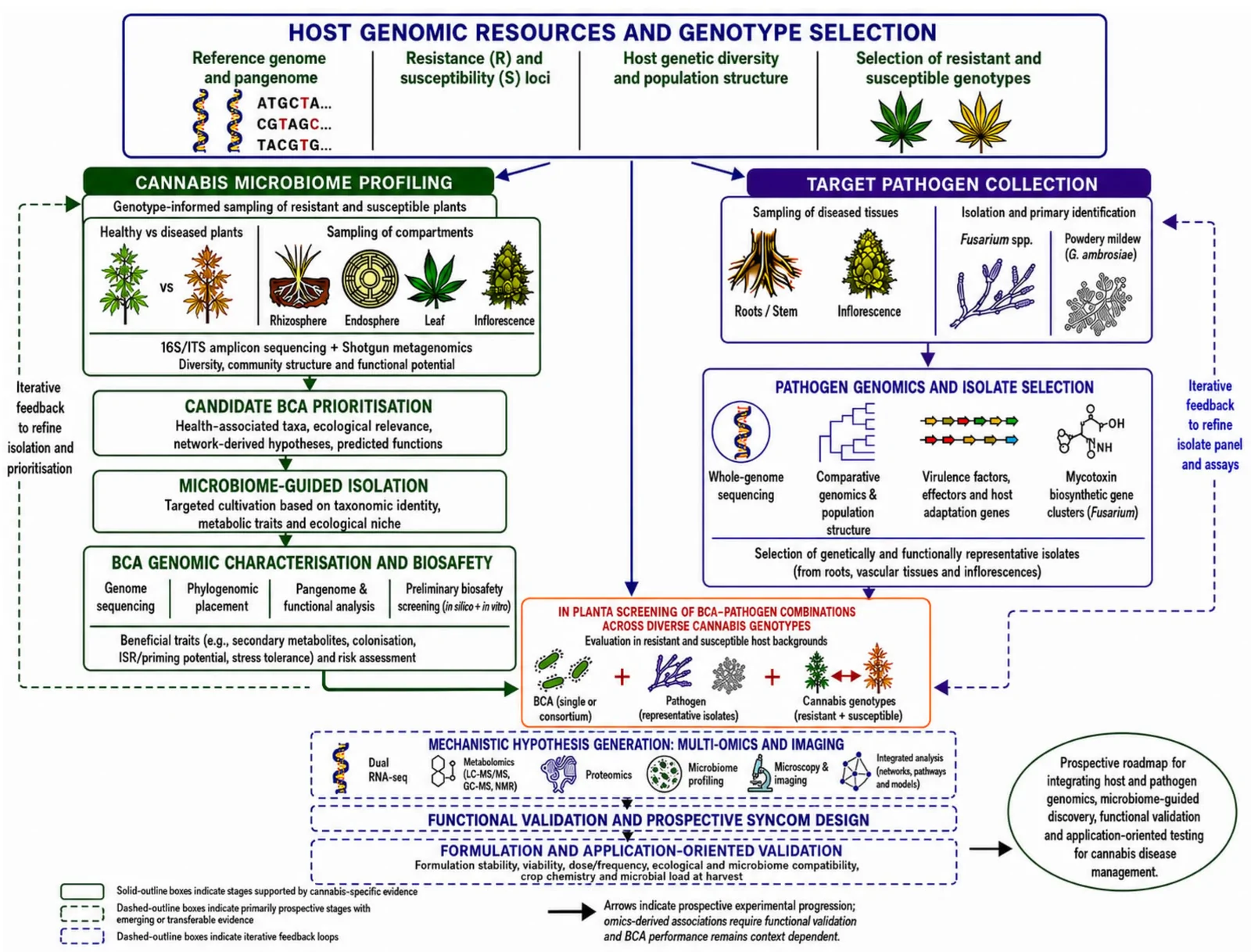
Prospective omics-guided roadmap for biological control research and BCA development in *Cannabis sativa*. The framework integrates established omics approaches around cannabis-specific experimental constraints, including host genetic and chemotypic diversity, pathogen diversity, compartment-specific microbiomes, crop-chemistry compatibility, and the microbiological quality of harvested inflorescences. Box styling reflects the availability and extent of evidence supporting individual components of each stage, rather than validation of the complete stage as part of an integrated cannabis-specific workflow. Cannabis-specific evidence ranges from direct BCA experiments to indirect or enabling genomic, microbiome and crop-quality resources, whereas predominantly prospective stages rely more strongly on methodological support from other plant pathosystems. Omics-derived associations require functional validation, while progression between stages remains conditional on reproducible efficacy across relevant biological and cultivation contexts.

The overall workflow of the proposed pipeline is summarised in Figure 2.

The following sections examine each step of the proposed pipeline in relation to the previously discussed interconnected bottlenecks, rather than treating individual omics technologies as ends in themselves. Evidence from other plant pathosystems is incorporated where relevant to provide conceptual and methodological support, while remaining clearly distinguished from cannabis-specific findings.

### 4.1. Cannabis Genomic Resources and Genotype Selection

Host genotype is a major biological component of the cannabis–pathogen–BCA interaction and should therefore be explicitly considered in experimental design [36,37,38,39,101,102]. It can influence disease susceptibility [36,37,38,39] and contribute to microbiome assembly [101,102], with potential consequences for the colonisation and activity of introduced BCAs. Genomic characterisation of plant material can therefore support the selection and stratification of host genotypes for microbiome profiling, pathogen challenge experiments and comparative *in planta* BCA evaluation. These resources should be regarded primarily as tools for defining host diversity and selecting informative experimental material.

The genomic resources available for *C. sativa* have expanded substantially during the last decade. Multiple chromosome-scale assemblies are now available for genetically diverse cannabis accessions, providing references for gene annotation, variant discovery and transcriptomic read assignment [24,25]. Genome-wide association studies (GWAS) have also demonstrated the potential of genetically diverse hemp panels for dissecting complex quantitative traits [103]. Using RAD-seq data from 123 accessions, Petit et al. [103] identified 16 QTL associated with fibre and cell-wall characteristics, together with candidate genes involved in carbohydrate and lignin metabolism. Although these traits are not directly related to disease resistance, the study illustrates more broadly how association panels and genome-wide marker data can resolve host genetic diversity for downstream experimental stratification.

More recently, Lynch et al. [104] generated a cannabis pangenome comprising 181 newly assembled and 12 previously published genomes representing 144 biological samples. This resource captures extensive sequence and structural variation that is not fully represented by a single reference genome and provides a framework for investigating copy-number and presence-absence variation in resistance, susceptibility and immune-related genes. The pangenome therefore offers a broader catalogue of variation against which experimental cannabis accessions can be compared. Population-genomic studies based on whole-genome resequencing, genotyping-by-sequencing and dense SNP datasets have been used to assess relatedness and population structure among cannabis accessions [105]. These resources can therefore help define genetic backgrounds relevant to disease susceptibility and microbiome composition, thereby supporting future studies of genotype-dependent variation in BCA performance.

PM currently provides the clearest example of how host genomics can guide genotype selection in cannabis disease research [36,37,38,39]. Mihalyov and Garfinkel [36] identified *PM1*, a single dominant locus conferring complete resistance to a tested *G. ambrosiae* isolate, and developed a co-segregating marker that can support the selection of resistant and susceptible plants. *PM1* was mapped to a genomic interval containing several genes encoding nucleotide-binding leucine-rich repeat (NLR) immune receptors, historically referred to as NBS-LRR proteins [36]. Several candidates encode proteins containing coiled-coil, NB-ARC and leucine-rich repeat domains [36,106], suggesting that *PM1* may involve intracellular effector recognition. However, the causal gene underlying *PM1* has not yet been identified, and a role for intracellular effector recognition remains to be experimentally confirmed [36,106].

Seifi et al. [39] mapped *PM2* to chromosome 9 using bulked-segregant analysis combined with RNA sequencing and showed that this dominant locus strongly restricts PM infection and sporulation. Resistance was associated with localised reactive oxygen species accumulation and a hypersensitive response occurring mainly in epidermal cells. The identification of *PM2* illustrates the value of combining genetic mapping, RNA-seq-derived SNP markers and histochemical characterisation to investigate disease resistance in cannabis.

Genomic resources have also enabled the investigation of candidate PM susceptibility genes. Genome-wide analysis of the *C. sativa MLO* family identified *CsMLO1* and *CsMLO4* as strong candidates for PM susceptibility because both belong to the dicot-associated clade V and are transcriptionally induced following *G. ambrosiae* infection [37]. Further support for the involvement of *CsMLO1* was obtained in the resistant genotype ‘FL 58’. Stack et al. [38] mapped a major-effect resistance locus close to *CsMLO1* and identified a 6.8 kb insertion in the resistant allele. Part of this insertion was incorporated into the transcript, resulting in alternative splicing and a premature stop codon. Molecular markers derived from the altered allele strongly predicted the phenotype, consistent with a recessive inheritance model. Nevertheless, direct functional validation would help determine the extent to which disruption of *CsMLO1* accounts for resistance.

Overall, *PM1*, *PM2* and the locus containing *CsMLO1* illustrate that PM resistance in cannabis may arise through both dominant resistance loci and a putative recessive loss-of-susceptibility mechanism [36,37,38,39]. Although these loci have not yet been systematically used in BCA studies, they provide genetically defined backgrounds for prospectively testing how microbiome composition and BCA-mediated protection vary, either independently of or in interaction with major-gene resistance.

In contrast to PM, the genetic basis of cannabis resistance to *Fusarium* remains at an earlier stage of characterisation [8,35]. Studies in other plant species indicate that resistance or tolerance may involve cell-wall reinforcement, regulation of reactive oxygen species, jasmonic acid-, ethylene- and salicylic acid-dependent signalling, receptor-like kinases and defence-related transcription factors [107,108,109]. For example, differential expression of genes involved in lignification and cellulose biosynthesis has been related to contrasting responses to *F. oxysporum* in banana [108]. In *Arabidopsis*, RFO1/WAKL22, which encodes a wall-associated kinase-like receptor, and the transcription factor ERF1 have been functionally implicated in resistance to *F. oxysporum* [110,111]. These pathways may therefore represent candidates for investigation in cannabis, although their specific roles remain unresolved [107,108,109,110,111]. Until cannabis resistance loci are more clearly defined, selection of informative material for *Fusarium* and other pathosystems can be guided by reproducible quantitative phenotyping combined with genome-wide genotyping. Genotypes displaying contrasting levels of symptom development, pathogen biomass, vascular colonisation, tissue damage or, where relevant, mycotoxin accumulation can subsequently be used for QTL mapping, GWAS and comparative transcriptomic analyses. This approach could identify resistance loci while simultaneously providing diverse host backgrounds for microbiome profiling and BCA discovery.

Within the proposed pipeline, host genomic characterisation addresses the first bottleneck by ensuring that BCA performance is evaluated across genetically defined cannabis backgrounds rather than inferred from a single genotype. Cannabis genomic resources have not yet been systematically used to explain or predict genotype-dependent variation in BCA efficacy, making this an explicitly prospective component of the framework. Once informative host backgrounds have been selected, microbiome profiling can be used to identify taxa linked to plant health and community features potentially relevant to disease suppression.

### 4.2. Microbiome-Informed Prioritisation and Prospective Discovery of Candidate BCAs in Cannabis

*Cannabis sativa* hosts diverse bacterial and fungal communities in the rhizosphere, root-associated compartments, internal vegetative and reproductive tissues, phyllosphere and seeds [99,112,113]. These communities provide a broad reservoir of microorganisms for BCA discovery across cannabis compartments and cultivation environments [99,112,113]. Plant compartment is a major determinant of microbiome composition and network organisation, while cultivation site, developmental stage and host genotype further contribute to community variation [101,102,112,113,114]. Among these factors, genotype differences warrant particular consideration when selecting donor plants and interpreting associations involving candidate taxa [102].

Within the proposed framework, the central challenge is not simply to describe microbial diversity in cannabis, but to determine which observed associations can be translated into experimentally supported BCA candidates. Microbiome-derived patterns are therefore used prospectively to prioritise taxa and cultivation targets, rather than being regarded as demonstrated predictors of effective BCAs; attribution of disease-suppressive function requires subsequent experimental validation.

To address this challenge, community profiling can broaden the range of taxa considered for subsequent isolation beyond those identified through conventional antagonism screening [115,116]. A disease-oriented discovery workflow can begin with controlled comparisons of healthy and diseased cannabis plants matched, as far as possible, for genotype, developmental stage, cultivation batch, substrate, irrigation, nutrient regime and environmental conditions. Sampling can then be adapted to the infection biology and affected plant compartment of the target pathogen. Root, crown and vascular diseases require examination of belowground and internal tissues relevant to pathogen establishment, whereas PM studies can compare asymptomatic, pre-symptomatic and symptomatic leaf surfaces. For grey mould, floral *Fusarium* diseases and other bud rots, relevant aerial tissues, particularly inflorescences and, where appropriate, leaves, can be sampled across developmental and infection stages. Where relevant, resistant and susceptible genotypes could provide an additional informative contrast; for PM-focused studies, this may include material differing at *PM1*, *PM2* or the genomic regions containing *CsMLO1* [36,37,38,39].

Marker-gene amplicon sequencing has been widely used for culture-independent characterisation of cannabis-associated bacterial and fungal communities [101,102,113,114]. It enables the analysis of large sample sets and can reveal compartment-specific taxa, core microbiome members and community shifts related to genotype, developmental stage or cultivation environment [101,102,113,114].

Cannabis microbiome studies illustrate the strong effects of both plant compartment and host background. Comeau et al. [101] used bacterial 16S rRNA gene and fungal ITS sequencing to follow rhizosphere, root-endosphere and phyllosphere communities in three cannabis chemotypes throughout commercial production, revealing pronounced spatial and temporal variation and cultivar-associated differences, particularly in belowground compartments. In field-grown hemp, Ahmed et al. [114] identified compartment-specific core ASVs and highly connected taxa in roots and rhizosphere soil sampled across six locations, providing candidate taxa for prioritisation and subsequent targeted recovery.

Wei et al. [113] profiled bacterial and fungal communities in bulk soil, rhizosphere, roots, stems, leaves and flowers of four hemp ecotypes grown under common conditions. Soil represented a major source of microorganisms for cannabis plants, while microbial-network complexity declined along the soil-root-shoot continuum. These findings reinforce the value of considering the source plant compartment when selecting microorganisms for subsequent isolation and functional assessment. Ahmad et al. [102] similarly identified genotype- and compartment-specific core taxa in field-grown industrial hemp, including members of *Pseudomonas*, *Bacillus* and *Sphingomonas*, providing candidates for targeted isolation and subsequent functional evaluation.

Cannabis amplicon studies also illustrate technical considerations arising from host-derived DNA. Wei et al. [113] used nested 16S rRNA gene amplification to reduce chloroplast amplification, whereas Ahmad et al. [102] applied compartment-specific primer sets and peptide nucleic acid clamps to minimise chloroplast and mitochondrial reads. These refinements improve the recovery of bacterial community signals from plant samples. Marker-gene sequencing is therefore well suited to broad comparative community profiling but provides limited species- and strain-level resolution [117]. Inferred functional profiles derived from marker-gene data are best interpreted as predictions and can be supplemented by direct measurements of community gene content or activity [118]. Because amplicon datasets describe relative community composition [119], disease-oriented studies can be further strengthened by independent measurements of pathogen and total microbial abundance. Pathogen-specific qPCR can complement community profiling by providing independent estimates of pathogen biomass, as illustrated by assays developed for the detection and quantification of *F. graminearum* and *F. sporotrichioides* in hemp flowers [120]. In a related disease-oriented context, Rietman et al. [121] examined changes in hemp leaf-associated microbial communities following fungal inoculation and fungicide treatment. Together, these approaches illustrate how pathogen quantification and microbiome profiling can be combined to examine community variation in relation to pathogen abundance and disease-management outcomes in hemp.

Among the cannabis compartments relevant to microbiome-guided BCA discovery, seeds represent an additional and potentially valuable reservoir of microorganisms [99,122,123,124]. Amplicon sequencing has revealed cultivar-dependent bacterial endophytic communities in commercially sourced hemp seeds [122]. Culture-dependent analyses have also characterised cultivable bacterial endophytes from seeds and two-week-old sprouts of the hemp cultivar ‘Futura 75’, identifying isolates with potential plant growth-promoting traits [123]. Experiments on cannabis seedlings further showed that the endosphere is assembled from both seed-borne and soil-derived bacteria and is influenced by genotype and soil source [124]. Culture-based analyses have recovered seed-transmitted *Bacillus* and *Paenibacillus* isolates capable of inhibiting seed-associated fungi, including *Fusarium* spp. [99]. Integrating seed microbiome profiling with targeted candidate selection may therefore help identify microorganisms adapted to early cannabis colonisation for subsequent isolation and evaluation of biocontrol activity and, where supported experimentally, potential application in seed coating or biopriming.

Building on the extensive use of 16S rRNA gene and ITS amplicon sequencing in cannabis [101,102,113,114], shotgun metagenomics can extend community profiling by improving taxonomic resolution, supporting the reconstruction of metagenome-assembled genomes and characterising community functional potential [125]. In other plant pathosystems, genome-resolved microbiome analyses have identified taxa and functions related to disease suppression and guided the experimental reconstruction of protective communities [126,127]. Applied to cannabis, shotgun metagenomics could identify candidate genes and biosynthetic pathways associated with antimicrobial production, siderophore biosynthesis, biofilm formation, nutrient competition and plant colonisation. Metagenomic analyses can also provide information on community resistomes and the occurrence of antimicrobial-resistance determinants in environmental microbial communities [128]. Despite this potential, disease-oriented shotgun metagenomics and genome-resolved functional analyses have not yet been systematically applied to BCA discovery in cannabis.

The detection of a biosynthetic gene cluster or other functional determinant indicates genetic capacity for a given function rather than demonstrated activity [125]. Whether the corresponding pathway is active can subsequently be investigated during cannabis colonisation or pathogen challenge. Genome-resolved analyses can therefore be complemented by metatranscriptomics to identify microbial genes expressed during pathogen invasion [129] and by metabolomics to detect chemical changes associated with microbial-community structure and function [130].

As a hypothesis-generating approach, network analysis can identify highly connected taxa and reveal co-occurrence structure potentially relevant to disease suppression [114,116]. However, network hubs should not be assumed to represent keystone taxa or functionally important BCAs, because inferred associations do not establish ecological dependence or a causal contribution to disease suppression. Network-derived features should therefore be used as one component of a broader prioritisation strategy rather than as stand-alone criteria for BCA selection. Taxa prioritised in this way require isolation, reintroduction and host-based efficacy testing before any keystone role or protective function can be attributed to them. Candidate selection can then draw on convergent evidence, including reproducible enrichment in healthy plants, negative associations with independently quantified pathogen biomass, membership in health-associated community modules and putative protective traits.

The cannabis microbiome studies reviewed here are predominantly descriptive [101,102,113,114], while experimental datasets demonstrating that microorganisms or microbial functions related to healthy plants contribute causally to reproducible disease suppression following reintroduction remain scarce.

In this context, combining community profiling with independent pathogen quantification and convergent evidence from health-associated community patterns provides a route from observational microbiome data to identified taxa for downstream functional testing. This addresses the bottleneck between descriptive associations and candidate recovery by identifying signals that warrant direct experimental investigation. Selected taxa can then be targeted for cultivation, followed by strain-level genomic and biosafety characterisation in the next stage of the framework.

### 4.3. Microbial Isolation, Genomic Characterisation and Biosafety

In this stage of the proposed pipeline, microbial candidates prioritised through microbiome profiling and related discovery approaches can undergo microbiome-guided cultivation to recover viable strains for experimental validation. Culture conditions can be selected according to the inferred taxonomic affiliation of targeted candidates, their predicted metabolic requirements, and their microhabitat of origin. Rather than relying only on a limited set of standard cultivation conditions, microbiome information can be used to identify taxa that are underrepresented or absent from the culture fraction and to guide the selection of alternative media and incubation conditions [131].

This stage addresses three limitations of association-based discovery: the absence of traceable cultured representatives of prioritised taxa, the uncertain relationship between genome-encoded capacity and experimentally observed phenotypes, and the need to explicitly assess safety determinants.

Cannabis-specific studies are beginning to integrate culture-dependent and culture-independent approaches. Lobato et al. [131], for example, compared bacterial isolates recovered from cannabis seeds with 16S rRNA gene amplicon profiles of the corresponding seed microbiome. By revealing which microbiome members were represented in the culture collection and which lacked closely related cultured representatives, this comparison highlighted gaps that could guide media optimisation, alternative incubation conditions and targeted isolation. However, whether microbiome-guided cultivation improves the recovery of candidates that subsequently demonstrate reproducible biocontrol activity compared with conventional culture-based isolation strategies has not yet been established in cannabis.

Once candidate microorganisms have been recovered as cultured isolates, taxonomic and genomic profiling can establish strain identity and provide a basis for downstream functional and safety assessment. 16S rRNA gene sequencing for bacterial isolates or ITS sequencing for fungal isolates can provide a preliminary taxonomic identification, whereas whole-genome sequencing can support higher-resolution strain identification and comparative genomic characterisation. For bacterial isolates, average nucleotide identity, genome-wide similarity metrics and phylogenomic placement can support species-level delineation, taxonomic assignment relative to reference genomes and comparison with metagenome-assembled genomes [132,133]. This progression from microbiome-informed cultivation to genome-based isolate analysis is well illustrated in a non-cannabis model by Bai et al. [134], who combined microbiome profiling with draft genome sequencing of approximately 400 leaf- and root-derived bacterial isolates from *Arabidopsis thaliana*, linking community patterns with genome-encoded functions and colonisation traits.

A comparable isolate resource in cannabis, linking microbiome signatures with cultured strains available for functional validation, would represent a valuable extension of this approach. Existing collections of cannabis-derived microorganisms and bacterial strain panels already provide useful starting material [18,99,135]. Cannabis seed-derived *Bacillus* and *Paenibacillus* isolates have shown antifungal activity against *Fusarium*, *Alternaria*, *Aspergillus* and *Penicillium*, whereas selected *Bacillus* and *Pseudomonas* strains have inhibited *B. cinerea* and other culturable cannabis pathogens [18,99].

Additional genomic resources are also available, including draft genomes of six *Pseudomonas* strains isolated from cannabis roots and stems, which provide material for comparative analyses of genes potentially related to host colonisation, plant growth promotion and biological control [135]. More detailed genome-based investigation is exemplified by *B. velezensis* LBUM279 and LBUM1082, and *B. subtilis* LBUM979 [18]. Their genome sequences refined taxonomic placement and revealed 18 biosynthetic gene clusters of potential biocontrol interest, while *in planta* assays showed that selected *B. velezensis* strains reduced grey mould severity on cannabis leaves [18]. By linking genomic information with disease-suppression phenotypes, this study provides a useful model for integrating genome-based characterisation with experimental BCA evaluation in cannabis.

Beyond taxonomic and functional analysis, genomic information should form part of the preliminary hazard identification and biosafety assessment required before candidate strains advance to later stages of the pipeline. Whole-genome screening should include known virulence determinants, toxin-biosynthesis pathways and clinically relevant antimicrobial-resistance genes [136], together with evaluation of mobile genetic elements that may facilitate the transfer of undesirable traits. In the same cannabis study, the genomes of the selected *Bacillus* strains were also examined for major toxin- and virulence-associated genes [18], illustrating how safety-related genomic information can be incorporated alongside functional testing. Cannabis-specific evidence nevertheless remains limited to a small number of genomically characterised candidate strains, and broader biosafety assessments combining genomic and phenotypic data have not yet been systematically implemented across cannabis BCAs. This gap is particularly relevant when candidate microorganisms belong to genera that include both beneficial and opportunistic strains, or when they are intended for application to cannabis inflorescences. In the latter case, viable cells or spores may persist on the harvested product and affect its microbiological quality, while microbial metabolites may raise additional product-safety concerns.

Importantly, genomic screening alone cannot establish strain safety. Genome-based hazard identification is constrained by incomplete functional annotation, strain-specific genetic variation and the possibility that unknown or poorly characterised determinants are not captured by current databases. The detection of a putative virulence, toxin, or antimicrobial-resistance determinant does not, by itself, establish its expression or phenotypic effect, but should be regarded as a safety signal requiring appropriate follow-up testing. Conversely, the absence of currently recognised hazardous determinants should not be interpreted as evidence of strain safety. Genomic screening therefore remains preliminary and cannot replace phenotypic, toxicological and application-specific assessment.

For strains capable of producing secondary metabolites or secreted proteins, safety assessment should also consider potentially undesirable compounds with known or suspected toxicological or other safety-relevant properties [18,136]. Where relevant, genome-based predictions can be complemented by targeted metabolomic, chemical or protein-level analyses to determine whether predicted compounds are actually produced under conditions relevant to the intended application [22,23,130].

Biosafety should therefore be initiated at this stage and implemented as a staged advancement criterion throughout subsequent validation, rather than as a single late-stage assessment. Before *in planta* testing, candidate strains should undergo strain-level taxonomic identification [132,133] and genome-based screening for major virulence, toxin, and clinically relevant antimicrobial-resistance determinants [18,136], together with appropriate preliminary phenotypic screening to exclude candidates presenting clear safety concerns. Candidates that subsequently demonstrate reproducible *in planta* efficacy can then undergo more extensive product-relevant assessment, including, where appropriate to the candidate taxon and intended use, antimicrobial susceptibility, haemolytic or cytotoxic activity and toxin or undesirable metabolite production [136], together with evaluation of strain persistence and residual microbial load on harvested tissues, particularly inflorescences [10,14].

In this context, BCA genomics contributes to the framework primarily by improving strain-level characterisation and traceability, identifying putative functional traits and supporting early hazard identification, while phenotypic evidence remains necessary for subsequent efficacy and safety assessment. Overall, this stage links microbiome-informed candidate discovery to traceable, genomically characterised cultured strains while embedding biosafety as an explicit criterion for candidate advancement.

### 4.4. Pathogen Genomics and Selection of Representative Isolates

Another major determinant of reproducible BCA performance is pathogen diversity. Once candidate BCAs have been taxonomically identified, genomically profiled and subjected to preliminary safety screening, pathogen genomics can be used to define representative challenge panels rather than relying on a single reference isolate.

Whereas BCA genomics is primarily used to detect genes potentially associated with beneficial traits and to screen for undesirable determinants, pathogen genomics can refine species identification, resolve population structure and evolutionary relationships, and identify candidate virulence determinants and secondary-metabolite biosynthetic potential. This distinction is important because pathogen isolates can differ genetically and phenotypically [35,40]. Although pathogen genomic diversity has not yet been linked to differential BCA susceptibility in cannabis, it provides a rationale for multi-isolate testing, while phenotypic information on pathogenicity, aggressiveness and host specificity remains essential for selecting biologically representative challenge isolates.

*Fusarium* diseases provide a particularly clear example, as cannabis-associated isolates span genetically and biologically distinct species complexes and cause diverse disease syndromes [8,35,40]. Chin et al. [40] generated genome sequences for five *Fusarium* species recovered from symptomatic greenhouse-grown cannabis plants in British Columbia: *F. graminearum*, *F. sporotrichioides*, *F. culmorum*, *F. proliferatum* and *F. oxysporum*. Comparative genomic analyses placed these isolates within established phylogenetic groups containing isolates from other crop hosts, while secondary-metabolite analyses revealed biosynthetic potential for several mycotoxins, including trichothecenes, fumonisins, zearalenone, beauvericin, and culmorin [40].

Together, this genomic and predicted biosynthetic diversity indicates that activity against a single *F. oxysporum* isolate provides only a limited assessment of BCA breadth and supports evaluation across representative *Fusarium* isolates and species relevant to the target disease complex. However, cannabis-specific datasets linking *Fusarium* genomic or population diversity to differential BCA susceptibility remain scarce. Where relevant to the target disease complex, panels could include multiple species or phylogenetic lineages and isolates recovered from different affected plant compartments.

For PM, the first genome of *Golovinomyces ambrosiae*, a major cannabis PM pathogen, was recently generated using a hybrid assembly combining long- and short-read sequencing [137]. The 155.2 Mb assembly contained 6995 high-confidence gene models and showed a high abundance of transposable elements [137]. Among the predicted proteins, 169 were classified as candidate effectors, including 14 sharing features with experimentally characterised effectors from other PM pathosystems [137]. This resource provides a basis for candidate effector analysis, pathogen transcript mapping and hypothesis generation concerning molecular processes involved in host colonisation and virulence. Population resequencing across geographical locations and sampling years could further characterise variation in candidate effectors and other virulence-associated loci and, when combined with infection assays on differential cannabis genotypes, help define distinct pathotypes. This integration is particularly relevant for resistance loci involving candidate NLR proteins, because resistance may depend on the recognition of corresponding pathogen avirulence effectors [36,106,137]. Combining pathogen genomic data with testing on cannabis genotypes carrying contrasting resistance or susceptibility alleles could therefore clarify host–pathogen specificity. Species-level identification is also important because both *G. ambrosiae* and *Podosphaera macularis* have been reported as PM agents of cannabis [137,138]. Whether host resistance and BCA efficacy are conserved across these PM species remains to be established.

A similar principle applies to genome-informed isolate selection in *B. cinerea*. A high-quality reference genome is available for the widely used isolate B05.10, providing a foundation for comparative genomics and transcriptomic read assignment [139]. In a broader, non-cannabis host study, Caseys et al. [140] demonstrated substantial variation in *B. cinerea* virulence and significant pathogen-strain × host-species interactions. In an infectivity matrix comprising 98 *B. cinerea* strains and 90 plant genotypes from eight host species, this interaction accounted for approximately 16% of the total variation in lesion area. GWAS further revealed a highly polygenic architecture of virulence and host specificity, with candidate loci distributed across the pathogen genome [140]. These findings provide a rationale for evaluating cannabis BCAs against multiple *B. cinerea* isolates, including strains recovered from different affected tissues, geographical origins and levels of aggressiveness, although cannabis-specific studies comparing disease suppression among genetically or phenotypically diverse *B. cinerea* isolates remain limited.

Genome resources are also available for additional cannabis pathogens. Zima et al. [51], for example, generated draft genomes for four *Neofusicoccum parvum* isolates recovered from diseased hemp plants and experimentally confirmed to be pathogenic on *C. sativa*. Their genomes ranged from 42.8 to 44.4 Mb and contained approximately 16,500 predicted genes [51]. These resources can support comparative analyses of genomic variation, secreted proteins and other candidate virulence-related traits relevant to cannabis stem canker and dieback [51]. They also illustrate the value of developing cannabis-specific pathogen genome collections even when reference genomes from other host-associated isolates are already available.

Compared with the pathogens discussed above, cannabis-specific genomic resources for other important root and crown rot agents, including *Globisporangium*, *Pythium* and *Rhizoctonia* spp., remain less developed. Expanding pathogen collections would strengthen the selection of representative challenge isolates and facilitate the analysis of pathosystems involving multiple taxa. The recent characterisation of a root and crown rot complex involving *Fusarium* and *Globisporangium* species in outdoor-grown cannabis [141] further illustrates the importance of considering multispecies disease complexes when constructing challenge panels.

No cannabis-specific evidence or consensus guideline currently defines a numerical minimum for the number of isolates required in BCA challenge panels. Nevertheless, microbiological validation frameworks developed for cannabis testing emphasise the need to represent within-target biological diversity rather than relying on a single isolate. For example, cannabis-specific AOAC validation guidance for *Aspergillus* detection requires inclusivity testing with multiple well-characterised strains covering the target species, including at least 10 strains per required *Aspergillus* species and a minimum of 50 target strains overall for method adoption. Although such criteria were developed for analytical method validation and cannot be directly translated into BCA efficacy testing, they reinforce the principle that biological variability should be explicitly incorporated. Accordingly, within the proposed framework, challenge panels should, where feasible, include multiple independent isolates spanning the major species or phylogenetic lineages represented in the target disease complex and should be expanded when distinct pathotypes, tissue sources, geographical origins or contrasting levels of aggressiveness are known. The importance of capturing such variability is further supported by evidence from other plant pathosystems showing isolate-dependent differences in pathogen aggressiveness, host specificity and responses to microbial antagonists [41,140].

By capturing biologically meaningful pathogen diversity for downstream testing, this stage addresses the first major bottleneck and reduces the risk that candidate performance is inferred from a single pathogen isolate or pathotype. Representative challenge panels can then provide the basis for comparative *in planta* evaluation in the subsequent stage of the pipeline.

### 4.5. Cannabis-Specific in Planta Validation and Mechanistic Characterisation

Candidate BCAs prioritised through microbiome profiling and subsequently isolated and genomically characterised can then be evaluated in *C. sativa* against the representative pathogen challenge panels defined in the preceding stage. This moves beyond preliminary in vitro evidence to assess host colonisation, persistence and disease suppression in the target host–pathogen context. This stage marks a point at which the three major bottlenecks identified above begin to converge. Comparative *in planta* testing addresses the reproducibility of BCA efficacy across relevant biological and environmental diversity; mechanistic multi-omics can identify molecular and community-level patterns, while functional experiments can test their causal relevance; and concurrent assessment of crop chemistry, microbial load and other safety-relevant endpoints helps determine whether effective candidates remain compatible with cannabis product-quality requirements.

Comparative assays can evaluate candidate BCAs across isolates represented within the pathogen challenge panel and genetically characterised cannabis genotypes, as host variation may influence disease susceptibility [36,37,38,39] and resident microbiome composition [101,102,124], with potential consequences for BCA establishment and activity.

A practical strategy for accounting for this variability is to use staged factorial designs rather than attempting to test all sources of variation simultaneously. Initial *in planta* experiments could evaluate candidate BCAs across a limited set of genetically contrasting cannabis genotypes and representative pathogen isolates under standardised environmental conditions, allowing BCA × genotype and BCA × pathogen-isolate interactions to be assessed. Candidates showing reproducible activity could subsequently be tested across the broader challenge panel and under contrasting substrates and cultivation environments to evaluate the robustness of efficacy and BCA × environment interactions. Randomised block designs can further account for cultivation-batch or experimental-replicate effects. Responses should be examined at the individual-isolate level rather than only as averages across the challenge panel. Isolate-specific loss of efficacy should be interpreted as evidence of a restricted activity spectrum rather than obscured by averaging across the panel. Such outcomes can help define the biological limits of a candidate BCA and, where appropriate, refine its intended target spectrum. This staged approach limits experimental complexity while allowing context-dependent variation in BCA performance to be identified before application-oriented validation.

Disease incidence and severity, plant survival, biomass and, where appropriate, yield-related traits can be assessed together with pathogen load and BCA abundance quantified using appropriately validated qPCR or dPCR assays. Selective re-isolation and microscopy can provide complementary information on viability, localisation and tissue colonisation.

Building on the preliminary biosafety assessment initiated in the preceding stage, product-relevant safety and crop-quality endpoints should be incorporated from the earliest *in planta* stages of BCA validation. For medicinal and high-cannabinoid cannabis, crop-chemistry advancement criteria should be predefined before the first *in planta* evaluation of candidate BCAs and should reflect the applicable product specifications and intended phytochemical profile, including total THC and CBD contents, the THC/CBD ratio and, where relevant, terpene composition. For standardised cannabis products, maintenance of a consistent chemical profile within predefined acceptable ranges is a core product requirement rather than merely an additional crop-quality endpoint. Consequently, a BCA-induced change in cannabinoid or terpene composition may compromise product consistency and compliance with the intended specifications, even when the candidate provides effective disease suppression. Such deviations should therefore be treated as a potential stop criterion in candidate advancement. Likewise, for BCAs intended for application to aerial tissues, residual viable BCA load on harvested inflorescences should be incorporated into candidate-advancement decisions as a potential stop criterion, rather than as a downstream quality check [10,14]. A strain may provide effective disease suppression yet remain unsuitable for commercial use if its persistence increases bacterial or fungal loads to levels that compromise the microbial purity of the final product or exceed the applicable microbiological specifications [10,14]. Candidates exceeding the applicable microbiological specifications at harvest or producing cannabinoid or terpene profiles outside predefined acceptable ranges should be excluded from further translational development, irrespective of disease-suppression efficacy. Early-stage validation should therefore evaluate BCA efficacy together with plant growth, inflorescence yield [100], cannabinoid composition [100,142], microbial load at harvest [10,14] and, where relevant, mycotoxins [13] or other undesirable microbial metabolites. Where BCA-derived metabolites or secreted proteins raise specific safety concerns, targeted chemical, metabolomic or protein-level analyses should also be performed on harvested inflorescences to determine whether these products remain detectable at harvest and, where relevant, during postharvest handling. These combined endpoints can help determine whether disease suppression is both reproducible and compatible with the requirements of the final cannabis product.

Ahmed et al. [142] showed by LC-MS/MS that microbial inoculants altered the concentrations of several phytocannabinoids across five cannabis cultivars, including CBDVA, CBDV, CBG, CBD and CBGA. Although this study was not conducted in a disease-control context, it demonstrates that microbial treatments can produce cultivar-dependent metabolic effects and illustrates why crop-chemistry profiling should accompany BCA validation. Untargeted and targeted metabolomics can therefore support early candidate-advancement decisions by monitoring relevant phytocannabinoids and other crop-quality metabolites [142] alongside disease suppression, while also assessing putative BCA-derived compounds and host defence metabolites [130].

At this validation stage, microbiome analysis shifts from taxon prioritisation to examining the community context of BCA establishment and activity. Amplicon sequencing or metagenomics can assess treatment-associated changes in the resident microbiome [101,121,125], although persistence of the introduced BCA should be confirmed using strain-resolved methods [117]. Where community-level responses implicate specific taxa, available cannabis-derived isolate resources can provide complementary strain-level material for targeted mechanistic follow-up [99,135,143]. Cannabis studies have shown that resident microbial communities vary with genotype, developmental stage, soil origin and cultivation context [101,124], supporting the possibility that BCA establishment and activity may also be context-dependent [101,124]. However, whether resident cannabis microbiomes causally influence BCA establishment or disease suppression has not yet been directly resolved. Accordingly, BCA-associated changes in microbiome composition should not automatically be interpreted as beneficial. Introduced strains may alter resident community structure and microbial interactions in ways that are unrelated, or potentially detrimental, to disease suppression and crop quality. Such changes should therefore be evaluated alongside BCA establishment and efficacy, particularly following repeated applications.

This potential influence of the resident microbiome on BCA establishment and activity can be tested experimentally by comparing BCA persistence and disease suppression in natural and microbiome-reduced substrates. In cannabis, soil sterilisation has already been shown to alter bacterial community assembly within the seedling endosphere [124], providing a rationale for using sterilised-versus-natural substrate comparisons to investigate microbiome-dependent effects on BCA performance. Such experiments should, however, distinguish effects caused by microbiome depletion from physicochemical changes induced by sterilisation. Evidence from other plant systems indicates that antimicrobial or heat treatments can substantially alter plant-associated microbial communities [144], providing an additional strategy for microbiome perturbation. Because such perturbations may also affect non-target microorganisms, substrate properties or host physiology, appropriate treatment controls and, where possible, microbial reconstitution should be included. Gnotobiotic or defined-community systems could provide controlled models for mechanistic perturbation experiments, in which plants are inoculated with known microbial assemblages to distinguish BCA-intrinsic effects from interactions with resident microorganisms and to enable targeted removal, add-back, and community-reconstitution analyses [127,145]. In these designs, BCA abundance, pathogen biomass, disease phenotype and microbiome composition should be quantified in parallel to determine whether changes in disease suppression are associated with altered BCA establishment or interactions with the resident microbial community.

Building on these phenotypic, product-quality and microbiome-context assessments, time-resolved multi-omics can be used to investigate molecular and community-level processes involving the host, pathogen, BCA and resident microbiome [22,23]. Genomic information generated during BCA characterisation can identify genetic potential for antimicrobial metabolite production, nutrient competition, tissue colonisation and host interaction, while transcriptomics and metatranscriptomics can determine which genes and pathways associated with these capacities are transcriptionally active *in planta* [22,125,129]. Metabolomics, proteomics and secretome profiling can complement these analyses by detecting antimicrobial compounds, enzymes, signalling molecules and defence metabolites [22,23,130]. In parallel, community profiling can assess treatment-associated restructuring of the resident cannabis microbiome, while quantitative tracking of pathogen and BCA abundance can determine whether protection is accompanied by altered colonisation dynamics. Combining these molecular and community-level measurements with disease phenotypes can reveal patterns consistent with direct antagonism, resource competition, induced resistance, and microbiome-mediated protection, thereby generating testable mechanistic hypotheses. Multi-omics integration is most informative when multiple data types are generated from matched samples and coordinated time points and interpreted at the level of shared pathways or candidate mechanisms rather than through direct feature-by-feature correspondence. Concordant signals can strengthen mechanistic interpretation, whereas discordant patterns should guide focused follow-up experiments. Causal attribution should not rely on multi-omics associations alone but requires functional validation through targeted perturbations such as gene inactivation or complementation, assays using purified metabolites, split-root experiments and removal, add-back or reconstitution experiments with defined microbial communities [19,22,23,127,145].

For the transcriptomic component of this framework, existing cannabis resources provide useful baselines for experimental design. Braich et al. [146] generated a comprehensive transcriptome atlas encompassing vegetative and reproductive tissues, male and female plant material and different stages of female flower and trichome development. Hu et al. [147] further characterised tissue-specific gene expression, alternative splicing, putative long non-coding RNAs and transcript-derived SNP and indel variation in wild cannabis. Together, these studies emphasise the importance of matching tissue, developmental stage and genotype when interpreting pathogen- or BCA-responsive transcriptional changes.

Balthazar et al. [15] provided an early cannabis-specific example of molecular analysis in a cannabis–pathogen–BCA system. The authors evaluated eight putative defence-related genes in cannabis leaves following infection with *B. cinerea*, of which ERF1, HEL, PAL, PR1, and PR2 were strongly and persistently induced at local infection sites [15]. The study also examined the effects of root-applied *Pseudomonas* and *Bacillus* strains on systemic defence-gene expression and grey mould development. Although enhancement of the selected defence markers and systemic disease reduction were not reproducibly detected under the tested conditions, the study provided candidate molecular markers and an experimental framework for investigating BCA-induced protection in cannabis.

More recently, Cale et al. [53] used time-resolved dual RNA sequencing of cannabis inflorescences infected by *S. sclerotiorum*, integrated with anatomical characterisation of the infection process. The study detected extensive transcriptional reprogramming in both organisms, including cannabis defence and signal-transduction responses and fungal processes related to redox regulation and sugar catabolism. Although no BCA was included, this experimental design provides a useful template for mechanistic biocontrol studies. Introducing a BCA into a comparable system could help determine whether disease suppression is associated with earlier or stronger activation of host defence pathways, reduced expression of fungal virulence and nutrient-acquisition genes, or both.

A further example of linking genomic predictions with phenotypic screening is provided by Kaddoura et al. [143], who characterised seven *Bacillus* endophytes isolated from *Chelidonium majus* seeds and six *Pseudomonas* endophytes isolated from cannabis roots and stems. Several *Bacillus* strains inhibited *F. oxysporum*, *F. graminearum* and *R. solani* AG3 in vitro, while their genomes contained biosynthetic gene clusters predicted to encode fengycin, surfactin, bacilysin, bacillibactin and other potentially antimicrobial products [143]. Although these findings do not constitute mechanistic validation, they provide a basis for subsequent *in planta* expression analyses, targeted metabolite profiling and functional testing of the predicted pathways.

At the data-infrastructure level, the platform developed by Mansueto et al. [148] integrates cannabis genome assemblies, annotations, genetic maps, expression datasets, metabolic profiles and sample metadata and supports cross-omics studies. This resource facilitates data reuse, integration and comparison. Coordinated experiments in which host, pathogen, BCA, microbiome and phenotypic measurements are generated from matched samples could provide the harmonised evidence needed for mechanistic analysis and, with increasing data availability, for the development and testing of predictive models of disease suppression.

Among the cannabis studies reviewed here, comparative BCA efficacy, strain-resolved tracking, mechanistic molecular profiling, resident-microbiome responses and product-quality outcomes have largely been investigated separately rather than within a single coordinated experimental framework. Addressing this fragmentation would allow reproducibility, mechanistic causality and product compatibility to be evaluated jointly and would provide a stronger basis for future experimental SynCom design.

### 4.6. Prospective Multi-Omics-Guided SynCom Design and Application-Oriented Validation

Among the studies identified in this review, no disease-suppressive SynCom has yet been experimentally validated for the cannabis pathosystems considered here. The framework outlined below should therefore be regarded as a prospective strategy informed by cannabis microbiome and BCA resources together with proof-of-concept from other plant systems, rather than as a workflow already demonstrated to be reproducible in cannabis. Accordingly, the detailed SynCom-design and testing recommendations presented below should be interpreted as methodological extrapolations derived primarily from cross-pathosystem studies, rather than as design rules already validated in cannabis.

Following *in planta* validation of individual BCA candidates, this stage has two related objectives. First, strains showing reproducible disease suppression, effective colonisation, and favourable preliminary safety and crop-quality profiles could, where biologically justified, provide a starting pool for SynCom development. Second, whether effective candidates are advanced individually or as components of candidate SynComs, they require application-oriented validation to determine whether biological efficacy can be maintained under conditions relevant to practical deployment. This stage therefore primarily addresses the third major bottleneck identified above, while prospective SynCom development also revisits the first and second bottlenecks at the community level by requiring robust disease suppression and experimental validation of emergent community properties.

Cross-pathosystem studies demonstrate approaches for microbial-community simplification and the experimental assessment of priority effects and individual-member contributions [149,150]. Genomic, microbiome and other omics-derived information generated during the preceding stages may assist the initial prioritisation of potentially complementary strains [4,151]. To complement experimental compatibility testing, co-occurrence networks can generate hypotheses about potentially compatible or mutually exclusive community members [101,102,114]. However, these inferred associations cannot independently establish strain compatibility, keystone function or disease-suppressive activity, all of which require experimental verification. In particular, iterative community reduction has been used to identify simplified consortia retaining relevant functional properties [149]. A disease-suppressive microbiome was reduced to a defined consortium while retaining protective activity [152], whereas integration of microbiome profiling, network analysis, bacterial genomics, comparative SynCom testing and host transcriptomics supported community optimisation in a different functional context [153]. These approaches remain to be validated in cannabis disease systems.

Prospective SynCom development in cannabis could prioritise individual strains showing reproducible disease suppression and effective colonisation of the target cannabis compartment, based on information generated during the preceding stages, including genomic potential, microbiome association, experimentally supported phenotypes, and preliminary safety characteristics. SynCom design should additionally consider functional complementarity, inter-strain compatibility and the possibility that community properties cannot be predicted from individual-strain performance alone. Initial pairwise and multi-strain assays can identify strong antagonism or competitive exclusion among candidate members, but compatibility should subsequently be confirmed *in planta* because interactions observed in culture may not reflect those occurring in the cannabis microbiome. Priority effects can be tested by varying the order and timing of strain introduction and quantifying each SynCom member using strain-specific qPCR or digital PCR [150]. SynCom performance should also be compared with that of the corresponding individual strains and simpler strain combinations. Leave-one-member-out and add-back experiments can help determine whether particular members are required for disease suppression and community stability [150]. Communities showing persistent competitive exclusion, unstable composition or loss of disease-suppressive activity should be simplified or reformulated before further advancement. Compatibility should also be reassessed in the presence of the resident microbiome, because interactions observed in simplified systems may not persist under application-relevant conditions [154]. Accordingly, experimental evaluation should focus specifically on community-level properties that are not captured by validation of individual BCAs, including member establishment and persistence, inter-strain interactions, the functional contributions of specific members, and effects on the resident microbiome. These assessments should also consider effects on beneficial indigenous taxa, functional redundancy and community metabolic outputs.

Candidate SynComs that demonstrate reproducible disease suppression in initial cannabis experiments could subsequently be evaluated across diverse cannabis genotypes, representative pathogen challenge panels, cultivation substrates and environmental conditions. Such validation is important because cannabis-associated microbial communities and inoculant effects on plant metabolism can vary with host genotype, tissue and developmental stage [101,124], while phytocannabinoid responses may also be cultivar-dependent [142]. However, ecological and functional stability observed under controlled conditions should not be assumed to persist during practical deployment. Changes in substrate, resident microbiome and cultivation environment may alter the context in which SynComs assemble and function [101,102,112,113,114,124]. Nutrient regime, irrigation, and fertigation should additionally be treated as application variables requiring direct experimental assessment.

Beyond biological robustness under cultivation conditions, application-oriented validation should address practical delivery performance. Treatment dose and frequency, and compatibility with other relevant crop-management and crop-protection practices, should be assessed [44,52,100]. Repeated applications should be evaluated for cumulative effects on resident microbiome composition and function. Environmental stressors relevant to the target plant compartment, including humidity and UV exposure, should be incorporated into practical testing. Across successive cropping cycles, omics-based monitoring can further assess whether SynCom composition, member activity, interactions with the resident microbiome and community-associated metabolic outputs remain consistent [154,155,156]. In parallel, formulation stability, post-storage viability and retention of functional activity should be evaluated [17,57,157].

During application-oriented validation, the harvest-stage microbial-purity criterion defined in Section 4.5 should be operationalised according to the regulatory and quality specifications applicable to the intended product and jurisdiction, because a single numerical threshold cannot be applied across the proposed framework. For example, for cannabis flower intended for inhalation by vaporisation, the European Pharmacopoeia criteria for non-sterile preparations for inhalation specify a Total Aerobic Microbial Count (TAMC) of 10^2^ CFU/g and a Total Yeast and Mould Count (TYMC) of 10^1^ CFU/g, corresponding to maximum acceptable counts of 200 and 20 CFU/g, respectively. Candidate progression should therefore be evaluated against the relevant limits for total microbial burden and specified objectionable microorganisms [14]. Accordingly, this stage should quantify both total microbial load and strain-specific BCA persistence from the final application through harvest and, where relevant, during postharvest handling [14]. Application dose, timing, target tissue and the interval between the final treatment and harvest should be optimised to retain disease protection while limiting residual BCA populations [14,17].

Advancement should ultimately integrate reproducible disease suppression, plant colonisation, ecological and functional stability where community-based interventions are considered, crop productivity and chemistry, microbiological quality at harvest, formulation performance and biosafety. The crop-chemistry and harvest-stage microbial-purity requirements defined during early *in planta* validation should remain explicit advancement criteria throughout application-oriented assessment. Failure to meet either requirement should prevent progression towards production-scale validation, irrespective of disease-suppression efficacy. These criteria should be reassessed across relevant genotypes, cultivation cycles and environmental conditions. Until a cannabis SynCom-development workflow has been experimentally validated, candidate communities should progress through iterative testing, simplification or reformulation and re-evaluation.

Further commercial translation would depend on jurisdiction-specific regulatory approval, manufacturing consistency, toxicological and residue-related safety requirements and cost-effectiveness relative to existing disease-management practices. Preliminary economic assessment should consider production and formulation costs, shelf life, storage and distribution requirements, application frequency, labour and equipment requirements, compatibility with existing cultivation practices and the expected reduction in disease-related crop losses. A biologically effective BCA or SynCom may remain commercially unsuitable if its implementation costs are disproportionate to the achievable disease-management benefit. Although full techno-economic analysis, product registration and commercialisation fall beyond the scope of the present omics-focused framework, comparative cost-effectiveness should be incorporated as a later-stage advancement criterion.

Table 3 provides an evidence map of representative studies and resources supporting each stage of the omics-guided pipeline described above. Evidence categories are not mutually exclusive. “Cannabis-specific direct evidence” refers to studies conducted in *C. sativa* or against cannabis-associated pathogens that evaluate BCA activity, disease suppression, BCA establishment or a BCA-associated mechanism. Because this category encompasses study designs providing different levels of support for BCA efficacy, the experimental system, such as in vitro antagonism, detached-tissue testing, controlled whole-plant testing or greenhouse evaluation, is specified alongside the evidence category. “Cannabis-specific indirect evidence” refers to cannabis studies that provide genomic, microbiome, pathogen, molecular or crop-quality information relevant to a framework component but do not directly test BCA efficacy or causal contribution to disease suppression. “Cross-pathosystem support” refers to methodological or mechanistic proof-of-concept obtained in other plant systems. “Conceptual proposal” refers to framework components proposed here as future directions that have not yet been experimentally demonstrated in the corresponding cannabis pathosystem. These evidence categories describe the relationship of each study or resource to a specific component of the proposed framework; they do not constitute a risk-of-bias assessment or a methodological-quality grading system. Accordingly, the terms “direct” and “indirect” should not be interpreted as indicating higher or lower study quality. Category assignments apply to the specific statement, resource or framework component represented in each row, rather than globally to an entire publication. Consequently, the same publication may provide direct evidence for one component and indirect or enabling evidence for another. Some overlap in cited studies with Table 1 and Table 2 is intentional rather than duplicative: Table 1 is organised around pathogen biology and its implications for BCA selection and delivery, Table 2 summarises study-level cannabis-specific evidence for individual BCA-pathogen combinations and levels of experimental support, whereas Table 3 recontextualises selected studies and enabling resources according to their position in the sequential pipeline, the evidentiary role they play and the principal knowledge gap remaining at each stage.

## 5. Discussion, Conclusions and Future Perspectives

Biological control represents a promising component of sustainable cannabis disease management, particularly because conventional chemical control may be constrained by the limited availability of fungicides registered for cannabis [7], pesticide-residue concerns and stringent product-quality requirements [7,14,158]. Nevertheless, available evidence remains fragmented across several complementary research areas, including plant pathology, microbiome research, microbial biotechnology and cannabis secondary metabolism. A recent analysis of the cannabis-bacterium literature identified distinct research themes encompassing plant growth promotion, modulation of specialised metabolites, antibacterial properties of cannabis-derived compounds and microbial disease suppression [27]. Bringing these areas together can help clarify how host genotype, pathogen diversity, resident microbiomes, BCA activity and crop chemical quality jointly shape disease-control outcomes. The framework proposed here therefore extends beyond simply adding omics technologies to conventional BCA screening by integrating multi-omics evidence into an iterative decision-making process linking microbial discovery, mechanistic investigation, and practical application.

The need for such an integrative framework becomes particularly evident when considering the strong context dependence of biological control. Cannabis genotypes may differ in disease susceptibility and microbial recruitment [36,37,38,39,101,102], while cultivar-dependent effects of microbial inoculation on phytocannabinoid profiles have also been reported [142]. Pathogen species and isolates can likewise vary in aggressiveness and host- or tissue-associated behaviour [35,40,140], while cross-pathosystem evidence indicates that susceptibility to microbial antagonists can also differ among *Fusarium* isolates [41]. Resident bacterial and fungal communities are also influenced by host genotype, plant compartment, developmental stage, seed and soil origin and cultivation conditions [101,102,112,113,114,124]. BCA efficacy can therefore be viewed as context-dependent, emerging from interactions among the host, pathogen, BCA and resident microbiome rather than as a fixed property of an individual strain. Potential non-target effects on resident beneficial taxa, community functional redundancy and microbial metabolic activity should also be considered, particularly following repeated applications or the introduction of multi-strain communities. Among the cannabis studies reviewed here, such cumulative community-level effects remain largely unresolved. Accordingly, reproducibility should be evaluated across biologically relevant host, pathogen and cultivation environments, with longitudinal measurements used where necessary to explain variation in treatment performance.

Beyond resolving the context dependence of BCA efficacy, a further priority is to connect omics-derived associations with experimentally supported mechanisms. Genome sequencing and comparative genomics can identify genetic potential for antimicrobial production, nutrient competition, tissue colonisation, and stress tolerance, while complementary molecular and functional experiments are required to determine whether the associated genes, pathways, or metabolites are active *in planta* and contribute to disease suppression [22,23,116]. Similarly, microbiome associations, co-occurrence networks, differentially expressed genes and metabolite correlations can generate mechanistic hypotheses whose functional relevance can then be experimentally tested [22,23,116]. Time-resolved factorial experiments combining appropriate host, pathogen, and BCA controls can help distinguish constitutive responses, treatment-induced effects, and processes specifically related to disease suppression.

Available cannabis molecular studies relevant to mechanistic biocontrol research illustrate both the insights provided by existing approaches and the need for broader investigation [15,53]. Balthazar et al. [15] directly examined defence-marker responses in a *C. sativa-B. cinerea*-BCA system, whereas the study by Cale et al. [53], although not including a BCA, provides a dual-transcriptomic template spanning disease progression that could be extended to three-way cannabis–pathogen–BCA interactions. Extending such designs with quantitative BCA and pathogen tracking, and targeted molecular measurements could provide evidence for evaluating direct antagonism, nutrient competition, host defence priming and microbiome-mediated protection. Candidate mechanisms would then require targeted functional validation, for example through microbial mutants, complementation, metabolite assays or community reconstitution [19,116].

Mechanistic understanding alone is not sufficient for the development of biological control strategies in cannabis, because disease suppression must ultimately be considered alongside crop performance, product quality and microbiological safety. This is particularly important in cannabis, where microbial treatments may influence not only plant health but also the chemical composition and quality attributes of the harvested product. Microbial inoculation can alter phytocannabinoid accumulation in a cultivar-dependent manner [142]. Broader crop-chemistry endpoints, including terpenes, phenolics, flavonoids and defence-associated metabolites, could also be monitored where relevant. Candidate treatments intended for application during cultivation should combine effective disease control with the maintenance of the desirable crop-chemistry profile, inflorescence quality, and acceptable microbiological quality [10,14,142]. For microbial BCAs, this requirement creates a specific trade-off: persistence sufficient to support protection during flowering may become undesirable if viable BCA populations remain high on harvested inflorescences [10,14]. Omics can support this assessment, but candidate advancement should ultimately depend on demonstrated disease suppression and product compatibility rather than on molecular profiles alone.

These principles can also inform the prospective development of synthetic microbial communities. Defined communities provide experimentally tractable systems for testing microbial interactions and community functions [116,127]. Studies in other plant systems have shown that disease-suppressive functions can be reconstructed in defined microbial communities [127] and retained following community simplification [152]. Accordingly, SynCom development for cannabis disease suppression remains a prospective objective rather than an established strategy. For cannabis, the key challenge is therefore not simply to assemble multi-strain inocula, but to determine whether minimal, experimentally tractable communities can combine reproducible protection with functional complementarity, inter-strain compatibility and ecological stability under application-relevant conditions [149,150,153]. Such an approach shifts the emphasis from maximising community complexity towards defining microbial consortia whose composition, activity and contribution to disease suppression can be experimentally resolved. Importantly, robust colonisation under controlled conditions does not necessarily imply ecological or functional stability under practical deployment. As experimental complexity increases, context-dependent changes may reorganise interactions among community members and compromise disease-suppressive function [101,102,112,113,114,124,149,150,153].

Once effective strains or communities have been identified, further application-oriented development will also depend on formulation and delivery. For bacterial components, particularly non-spore-forming strains, maintaining cell viability during formulation and storage is an important consideration [157].

Progress towards reproducible biological control across experiments and cultivation environments will also depend on progressively larger, well-annotated and interoperable data. The use of authenticated cannabis genotypes, taxonomically and genomically profiled pathogen isolates and well-characterised BCA strains, together with appropriate controls and detailed metadata describing plant tissue, developmental stage, substrate, environmental conditions, inoculation timing and formulation, would strengthen cross-study comparisons. Integrating molecular measurements with consistently collected phenotypic and agronomic information could help relate pathogen biomass, BCA establishment, and microbiome responses to crop chemistry and agronomic performance [4]. Cannabis-specific bioinformatics infrastructures may further facilitate data integration, reuse and comparative analysis and support more consistent organisation of sample metadata [148]. As sufficiently large harmonised datasets accumulate across independent studies and cultivation environments, predictive models could be developed and tested more rigorously. Public deposition of omics data, strain genomes, metadata and reproducible bioinformatic workflows would further support transparency and reuse across cannabis biocontrol research.

Taken together, the principal priorities for advancing cannabis biological control are therefore not the accumulation of additional descriptive omics data per se, but the resolution of three interconnected bottlenecks: translating candidate activity into reproducible *in planta* efficacy across biological and environmental diversity; establishing causal links between omics-derived associations and BCA function; and demonstrating that effective candidates remain compatible with biosafety, crop chemistry, microbiological quality and formulation requirements under application-relevant conditions. Addressing these bottlenecks will determine whether omics-guided approaches can provide a practical advantage over conventional empirical screening rather than simply increasing analytical complexity.

Against this background, cannabis-specific evidence currently supports the potential of selected microbial BCAs against several fungal and oomycete pathogens [18,19,20,21], but remains too limited and heterogeneous to establish a validated omics-guided pathway from BCA discovery to application. The framework proposed here should therefore be regarded as a prospective roadmap for structuring future research, integrating host and pathogen diversity, microbiome-informed candidate selection, functional validation of omics-derived signals and application-oriented assessment under relevant cultivation conditions. At present, however, most mechanisms proposed in cannabis-specific BCA systems remain hypotheses or associations and have not undergone perturbation-based functional validation. Additional cannabis-specific studies will therefore be required to determine whether this integrated approach can reproducibly support the development of effective BCAs with experimentally substantiated mechanisms and, prospectively, of SynComs compatible with cannabis crop quality, microbiological safety and application requirements.

## Figures and Tables

**Table 1:** Major fungal and oomycete pathogens of *Cannabis sativa*, their principal disease characteristics, and implications for BCA selection and delivery.

Pathogen or Pathogen Group	Principal Disease(s)	Main Plant Compartments Affected	Predominant Risk Period or Cultivation Context	Major Symptoms and Consequences	Implications for BCA Selection and Delivery	References
*Fusarium* *oxysporum species complex*	Damping-off Root and crown rot Vascular wilt	Seeds Roots and crown Stem base Vascular tissues	Propagation Vegetative growth Greenhouse production Hydroponic production	Poor emergence Root decay Vascular browning and chlorosis Stunting, wilting and plant collapse	Seed, root, rhizoplane or endophytic colonisation Persistence in the substrate Preventive application before vascular colonisation	[8,30]
*Fusarium solani species complex and other root-associated Fusarium spp.*	Root rot Crown rot Stem rot	Roots Crown Lower stem	Propagation Vegetative growth	Root discolouration Tissue necrosis Crown lesions Reduced plant vigour	Multiple species and isolates in challenge panels Representation of biological and pathogenic diversity Root- and crown-targeted BCA activity	[8,31]
*Fusarium spp.* *infecting cannabis* *inflorescences*, *including* *F. graminearum*, *F. sporotrichioides*, *F. proliferatum and* *F. avenaceum*	Bud rot Inflorescence blight	Flowers Inflorescences	Flowering Harvest Postharvest handling	Inflorescence necrosis Loss of commercial quality Potential mycotoxin accumulation	Persistence on reproductive tissues Minimal residual microbial contamination Absence of undesirable metabolite production Monitoring of pathogen biomass and mycotoxins	[8,10,13,33]
*Golovinomyces spp.*, *particularly* *G. ambrosiae*	Powdery mildew (PM)	Leaves Stems Floral bracts Inflorescences	Vegetative growth Flowering Indoor/greenhouse production	White epiphytic colonies Loss of plant vigour Reduced leaf and inflorescence quality	Screening on living host tissue Effective phyllosphere establishment and persistence Performance under fluctuating aerial- tissue conditions	[7,44,52]
*Botrytis cinerea*	Grey mould/bud rot Leaf blight Damping-off	Leaves Stems Flowers Dense inflorescences	Flowering Harvest and postharvest High-humidity conditions	Tissue browning and necrosis Soft rot Sporulation Rapid loss of inflorescence quality	Persistence at foliar/floral infection sites Separate evaluation of direct antagonism and root-induced systemic protection	[10,11,15,18,19]
*Pythium spp.*, *including* *P. myriotylum*, *P. dissotocum and* *P. aphanidermatum*	Damping-off Root and crown rot	Roots Crown	Propagation Hydroponic cultivation Water-saturated substrates	Root browning and decay Stunting Plant mortality	Root- and substrate-adapted BCAs Preventive establishment before pathogen exposure Integration with water-management practices	[9,20]
*Rhizoctonia solani*	Root rot Sore shin	Roots Lower stem	Seedling establishment	Root necrosis Reddish-brown lower-stem lesions Wilting Reduced plant vigour	Root- and substrate-adapted BCAs Preventive application during seedling establishment Targeting of root and stem-base infection sites	[49]
*Sclerotinia* *sclerotiorum and* *S. minor*	Stem canker Crown rot Inflorescence infection	Crown Stems Inflorescences	Field/greenhouse cultivation Flowering	Water-soaked lesions Stem collapse and necrosis Floral decay	Colonisation of affected tissues Direct antagonistic activity Mechanistic assessment using time-resolved molecular and anatomical approaches	[12,50,53]
*Sclerotium rolfsii*	Southern blight Crown/stem-base rot	Crown Stem base Soil-contact tissues	Warm conditions High-humidity conditions	Necrosis Wilting Mycelial growth Sclerotium formation	Rhizosphere- or crown-colonising BCAs Antifungal metabolite production Further cannabis-specific *in planta* validation required	[21]
*Neofusicoccum parvum*	Stem canker Dieback	Stems Branches	Field-grown hemp	Stem cankers Progressive terminal dieback	Genome-resolved pathogen collections Evaluation of isolate diversity Identification of BCAs active against emerging stem pathogens	[51]

**Table 2:** Cannabis-specific evidence for microbial biological control against fungal and oomycete pathogens.

BCA, Consortium or Microbial Product	Target Pathogen or Disease	Experimental System	Application or Treatment	Main Reported Outcome	Level and Type of Evidence	Reference
*Fusarium* spp. and *Pythium* spp. root/seedling disease targets						
Consortium of *Burkholderia ambifaria*, *Gluconacetobacter* *diazotrophicus* and *Herbaspirillum* *seropedicae*	Isolate designated by the authors as *F. oxysporum* f. sp. cannabis	In vitro assays Pre- and post-emergence cannabis assays	Bacterial consortium Assays targeting seedling disease	Pathogen inhibition Reduced disease severity	In vitro antagonism Controlled *in planta* disease-suppression evidence	[98]
Seed-borne *Bacillus* and *Paenibacillus* isolates	Seed-associated fungi *Fusarium* spp. included	Isolation assays In vitro antagonism assays	Endophytes recovered from cannabis seeds or seedlings	Several isolates inhibited seed-associated fungi Selected *B. velezensis* and *Paenibacillus polymyxa* isolates active against all fungal isolates tested	In vitro antagonism only	[99]
Commercial microbial products containing *Bacillus*, *Gliocladium* or *Trichoderma* spp.	*F. oxysporum* *Pythium myriotylum*	Controlled cannabis propagation assays	Preventive application before pathogen inoculation	Lalstop, RootShield, Asperello and Stargus reduced disease caused by *F. oxysporum* RootShield and Lalstop were the most effective treatments overall against *P. myriotylum*	Controlled *in planta* disease-suppression evidence	[20]
*Botrytis cinerea*/grey mould and related aerial fungal targets						
Selected *Bacillus* and *Pseudomonas* strains	*Botrytis cinerea* Other culturable cannabis fungal pathogens	In vitro antagonism assays Cannabis leaf assays	Direct application of selected bacterial isolates	Inhibited fungal growth Reduced grey mould severity	In vitro antagonism Controlled *in planta* disease-suppression evidence	[18]
*Pseudomonas protegens* Pf-5 and antibiotic-deficient derivatives	*Botrytis cinerea*	Cannabis leaf disease assay Mutant analysis	Leaf application 48 h before pathogen inoculation	Pyoluteorin and 2,4-diacetylphloroglucinol contributed substantially to disease suppression	Mechanistically supported controlled *in planta* evidence	[19]
Root-applied *Pseudomonas* and *Bacillus* strains, individually or in consortia	Foliar grey mould caused by *B. cinerea*	Controlled cannabis assays Targeted RT-qPCR	Root inoculation before foliar pathogen challenge	Systemic disease reduction not reproducibly detected Enhancement of selected defence markers not reproducibly detected	Controlled *in planta* mechanistic experiment No reproducible disease suppression detected	[15]
*Trichoderma asperellum*	*B. cinerea* *S. sclerotiorum*	In vitro confrontation assays Detached cannabis inflorescences	Spray application to detached inflorescences 48 h before *B. cinerea* inoculation	Inhibited both pathogens in vitro Reduced grey mould development on cannabis inflorescences	In vitro antagonism Detached-inflorescence disease-suppression evidence	[10]
Powdery mildew						
Rhapsody ASO (*B. subtilis* QST 713), Stargus (*B. amyloliquefaciens* F727), and Actinovate (*Streptomyces lydicus* WYEC 108)	Powdery mildew	Repeated indoor whole-plant trials Susceptible cannabis	Repeated foliar application	Rhapsody ASO and Stargus consistently reduced PM development Actinovate showed lower and less consistent efficacy	Indoor whole-plant disease-suppression evidence	[44]
Commercial formulations based on *B. amyloliquefaciens*	*Golovinomyces ambrosiae*	Greenhouse assays Susceptible hemp cultivars	Foliar application	Significant reduction in PM severity	Greenhouse whole-plant disease-suppression evidence	[52]
*Sclerotium rolfsii*/southern stem rot						
Endophytic isolates of *Trichoderma lixii*, *Lasiodiplodia brasiliensis* and *Macrophomina phaseolina*	*Sclerotium rolfsii*	Dual-culture assays Enzymatic analyses Microscopy Metabolite analyses Greenhouse pot assays	Inoculation of fungal endophytes before pathogen challenge	Inhibition of pathogen growth Substantial reduction in southern stem rot severity Strongest protection provided by *L. brasiliensis*	In vitro mechanistic evidence Greenhouse *in planta* disease-suppression evidence	[21]

**Table 3:** Evidence map, enabling resources and knowledge gaps across the sequential omics-guided framework for biological control in *Cannabis sativa*.

Omics Resource or Approach	Current Evidence and Contribution to the Framework	Evidence Category and Supporting References	Main Limitation or Knowledge Gap
Step 1. Host genomic characterisation and genotype selection			
Reference genomes Chromosome-scale assemblies Pangenomics Population-genomic analyses	References for gene annotation, variant discovery, population analyses and transcriptomic read assignment across genetically diverse accessions Support genotype authentication and assessment of genetic diversity Enable selection of informative plant material for downstream BCA experiments	Cannabis-specific indirect evidence [24,25,104,105]	Not yet systematically used to investigate or explain genotype-dependent variation in BCA efficacy Current contribution to biological control is primarily enabling
RAD-seq genotyping GWAS Genome-wide marker analysis	Genome-wide marker datasets and association panels demonstrate the feasibility of characterising complex genetic variation in cannabis QTL for fibre and cell-wall traits illustrate how genetically informative material can be selected for downstream experiments	Cannabis-specific indirect evidence [103]	Available association studies were not designed to investigate disease resistance or BCA responsiveness Direct mapping of genetic determinants of BCA efficacy remains to be performed
Genetic mapping Marker development for PM resistance	*PM1* and *PM2* provide genetically defined resistant and susceptible cannabis backgrounds These backgrounds can be incorporated into comparative BCA experiments	Cannabis-specific indirect evidence [36,39]	The loci establish variation in host resistance They have not yet been used to demonstrate genotype-dependent differences in BCA efficacy
Genome-wide MLO analysis Expression analysis Genetic mapping	*CsMLO1* and *CsMLO4* identified as candidate PM susceptibility genes Additional evidence links an altered *CsMLO1* allele to resistance These loci provide genetically defined backgrounds for investigating host-dependent BCA effects	Cannabis-specific indirect evidence [37,38]	Contribution to BCA-mediated protection remains prospective No direct association between MLO variation and BCA performance has been established
NLR-associated genomic resources Immune-resistance resources	Cannabis studies and syntheses identify immune-receptor-rich regions and candidate NLR-associated mechanisms involved in PM resistance These resources provide a molecular framework for investigating BCA-induced host responses	Cannabis-specific indirect evidence [6,36,106]	Candidate immune mechanisms remain incompletely resolved They have not been directly linked to BCA-mediated protection
Step 2. Disease-oriented microbiome profiling and candidate prioritisation			
16S rRNA gene amplicon sequencing ITS amplicon sequencing	Cannabis microbiome studies show strong effects of plant compartment, genotype, developmental stage, soil source and cultivation environment Datasets identify compartment- and genotype-associated taxa for cultivation and functional assessment	Cannabis-specific indirect evidence [101,102,112,113,114]	Differential abundance, co-occurrence and network centrality indicate associations, not causal disease-suppressive functions Network analysis is a hypothesis-generating approach, rather than a strategy providing criteria for BCA selection Relative abundance does not necessarily reflect absolute microbial abundance
Seed and seedling microbiome profiling Culture-dependent characterisation	Seed and seedling studies reveal cultivar-dependent endophytes and contributions of seed- and soil-derived microorganisms to community assembly Seed-transmitted *Bacillus* and *Paenibacillus* isolates provide cultivable candidates for subsequent functional assessment	Cannabis-specific direct evidence -in vitro antagonism only [99] Cannabis-specific indirect evidence -seed microbiome characterisation [99,122,123,124]	Seed transmission, community association or in vitro antagonism alone does not demonstrate disease protection *in planta* Candidate strains require reintroduction and disease-suppression assays
Shotgun metagenomics MAG reconstruction Metatranscriptomics Metabolomics	Genome-resolved approaches can improve taxonomic resolution and identify community functional potential Metatranscriptomics can provide information on transcriptional activity, whereas metabolomics can characterise associated chemical phenotypes Other pathosystems demonstrate potential for identifying disease-associated functions and reconstructing protective communities	Cross-pathosystem support [125,126,127,128,129,130] Conceptual proposal	Disease-oriented shotgun metagenomics and genome-resolved functional analysis have not yet been systematically applied to cannabis BCA discovery Detection of genes or biosynthetic pathways indicates potential rather than demonstrated activity
Step 3. Targeted cultivation and isolation of potential BCA candidates			
Prospective microbiome-guided cultivation linked to community profiling	Comparison of cultured and uncultured fractions of cannabis seed microbiomes revealed taxa absent from conventional isolate collections These gaps can guide optimisation of cultivation conditions Comparable microbiome-to-isolate strategies have been developed in other plant systems	Cannabis-specific indirect evidence [131] Cross-pathosystem support [134]	It remains untested whether microbiome-guided cultivation systematically improves the recovery of microbial candidates that subsequently demonstrate reproducible disease suppression, relative to conventional isolation and antagonism-based screening
Step 4. Strain-level genomic characterisation and preliminary safety screening of candidate BCAs			
Whole-genome sequencing Comparative genomics Phylogenomics Biosynthetic gene-cluster prediction	Genome analyses of *Bacillus* and *Pseudomonas* strains used in cannabis-focused research refined taxonomic identity Revealed genome-encoded traits potentially associated with antimicrobial production, plant colonisation and other beneficial functions Genome screening of selected cannabis-associated *Bacillus* strains assessed major toxin- and virulence-associated determinants, providing preliminary safety information.	Cannabis-specific indirect evidence -genome-based strain characterisation [18,135,143]	Genomic potential does not establish expression or contribution to disease suppression Predicted functions require phenotypic and mechanistic confirmation The absence of known hazardous determinants does not establish safety or replace phenotypic, toxicological and application-specific assessment
In silico screening of antimicrobial-resistance determinants Virulence-associated determinants Toxin-associated determinants	Genomic screening can identify undesirable determinants Supports preliminary identification of currently recognised genomic safety concerns	Cannabis-specific indirect evidence - preliminary genome-based safety screening [18,143] Conceptual proposal - informed by regulatory guidance [136]	Genomic screening alone cannot establish strain safety Additional evaluation required for phenotypic antimicrobial susceptibility, toxin production, cytotoxicity or haemolysis where relevant Mobile genetic elements, persistence on harvested tissues and contamination risk also require assessment
Step 5. Pathogen genomic characterisation and representative challenge-panel selection			
Whole-genome sequencing Comparative genomics of cannabis-associated *Fusarium*	Genomic analyses reveal substantial phylogenetic and secondary-metabolite diversity among cannabis-associated *Fusarium* species Variation includes mycotoxin biosynthetic potential	Cannabis-specific indirect evidence [35,40]	Direct cannabis-specific evidence linking pathogen genomic variation to differential BCA susceptibility is currently lacking
Hybrid genome assembly Effector prediction in PM pathogens	A *Golovinomyces ambrosiae* reference genome supports transcript mapping and candidate-effector analysis Provides a resource for future integration with genetically defined cannabis resistance loci	Cannabis-specific indirect evidence [137]	Population genomic variation and pathotype structure remain largely unexplored Their potential relationship with BCA performance is also largely unexplored
Whole-genome sequencing of emerging cannabis pathogens	Genome sequences of cannabis-derived *Neofusicoccum parvum* isolates extend pathogen genomic resources beyond the major cannabis diseases Enable future comparative analyses of isolate diversity	Cannabis-specific indirect evidence [51]	Resources have not yet been experimentally linked to BCA susceptibility or genome-informed BCA selection
Comparative genomic selection Taxonomic selection Phenotypic selection of challenge isolates	Cannabis pathogens show substantial taxonomic and biological diversity Reference-genome and comparative-genomic resources from other pathosystems provide a basis for genome-informed isolate comparison Other plant systems demonstrate isolate-dependent differences in aggressiveness and susceptibility to microbial antagonists Together, these observations support representative multi-isolate challenge panels	Cannabis-specific indirect evidence [35,40,51,137,138,141] Cross-pathosystem support [41,139,140] Conceptual proposal	Genome-informed challenge panels have not yet been systematically established for cannabis biological control Genomic distance cannot currently be assumed to predict differential BCA susceptibility No evidence-based numerical minimum for panel size has been established Multi-isolate efficacy should be demonstrated before candidate BCAs progress to later-stage mechanistic or application-oriented evaluation.
Step 6. Comparative *in planta* BCA evaluation and product-quality assessment			
Disease phenotyping BCA/pathogen quantification Microscopy Microbial monitoring	Selected bacterial and fungal BCAs and commercial microbial products have suppressed disease in controlled cannabis assays Evidence includes whole-plant, greenhouse and detached-tissue systems Detailed study-level efficacy evidence is presented in Table 2	Cannabis-specific direct evidence -controlled seedling and propagation assays [20,98], controlled cannabis leaf assays [18,19], detached-inflorescence assays [10], indoor whole-plant assays [44], and greenhouse whole-plant or pot assays [21,52]	Evidence remains concentrated on relatively few BCA-pathogen-genotype combinations In vitro antagonism does not necessarily predict *in planta* efficacy Reproducible performance across diverse and application-relevant cultivation conditions remains insufficiently demonstrated
Targeted metabolomics LC-MS/MS of cannabis chemical traits	Microbial inoculation can alter phytocannabinoid accumulation in a cultivar-dependent manner Demonstrates that microbial treatments can influence commercially important crop chemistry Supports cannabinoid and broader crop-chemistry profiling as an early criterion for BCA advancement	Cannabis-specific indirect evidence [142]	Available evidence was not generated in a disease-control context Compatibility between disease suppression and maintenance of desired cannabinoid and broader crop-chemistry profiles remains insufficiently characterised
Harvest-stage microbiological quality assessment Harvest-stage chemical quality assessment	Cannabis-specific studies identify potential risks from pathogen-derived mycotoxins Microbial inoculation can affect crop-related traits and phytocannabinoid composition, indicating that BCA evaluation should consider plant performance and crop chemistry alongside disease suppression Cannabis-specific literature also highlights jurisdiction-dependent product-safety constraints, including requirements concerning pesticide residues Together, these findings support the incorporation of harvest-stage chemical, microbiological and product-quality endpoints into BCA evaluation. Failure to meet predefined crop-chemistry requirements or applicable microbiological specifications at harvest constitutes a potential stop criterion irrespective of disease-suppression efficacy.	Cannabis-specific indirect evidence - harvest-stage microbiological-quality and product-safety considerations [10,13,14,40,158]; crop-performance and crop-chemistry considerations [100,142] Conceptual proposal	Residual viable BCA populations at harvest have rarely been quantified in cannabis biocontrol studies Relationships among application timing, BCA persistence, disease suppression and compliance with product microbiological specifications remain poorly characterised
Step 7. Multi-omics-supported mechanistic investigation and functional validation			
Targeted RT-qPCR Microbial mutant analysis	Limited cannabis-specific studies have combined targeted host-response analyses or microbial genetic perturbation with *in planta* disease assays to investigate BCA-associated mechanisms. Detailed study-level mechanistic evidence is summarised in Table 2.	Cannabis-specific direct evidence -controlled *in planta* disease-suppression and host-response analysis [15] Cannabis-specific direct mechanistic evidence - controlled *in planta* microbial-mutant analysis [19]	Direct mechanistic evidence is restricted to very few BCA-pathogen systems Expression of selected defence genes alone cannot establish defence priming or induced resistance
Time-resolved dual RNA sequencing Anatomical characterisation	Dual RNA-seq of the *C. sativa*-*S. sclerotiorum* interaction revealed extensive transcriptional reprogramming in host and pathogen Provides a cannabis-specific experimental model that could be extended to BCA-containing systems	Cannabis-specific indirect evidence [53]	The study did not include a BCA Its relevance to biological control is therefore methodological and enabling rather than direct
Baseline tissue-resolved cannabis transcriptomics Developmental-stage-resolved cannabis transcriptomics	Transcriptomic atlases document extensive constitutive variation among tissues, developmental stages and genotypes Provide reference information for distinguishing BCA- or pathogen-induced responses from baseline transcriptional variation	Cannabis-specific indirect evidence [146,147]	Datasets were not generated in BCA experiments They provide experimental context rather than direct mechanistic evidence
Integrated genomics Transcriptomics Metabolomics Proteomics Secretomics Microbiome analysis	Cannabis-specific omics resources and broader methodological studies support integration across host, pathogen, BCA and resident microbiome layers Integrated measurements can generate hypotheses concerning antagonism, nutrient competition, host-mediated protection and microbiome-mediated effects	Cannabis-specific indirect evidence [4,151] Cross-pathosystem support [22,23,125,129,130] Conceptual proposal	Omics associations and agreement across molecular layers do not establish causality Discordant layers may reflect different temporal, spatial or regulatory processes and require experimental resolution
Functional testing of omics-derived hypotheses	Targeted perturbations, microbial mutants, complementation, metabolite supplementation and microbial-community reconstitution can test mechanisms inferred from omics data Limited cannabis-specific examples exist, with broader methodological support from other systems	Cannabis-specific direct mechanistic evidence -controlled *in planta* microbial-mutant analysis [19] Cross-pathosystem support [22,23,116] Conceptual proposal	Such experiments remain uncommon in cannabis They are required before candidate pathways or community associations can be assigned causal roles in disease suppression
Integrated cannabis multi-omics data infrastructure	Cannabis bioinformatics infrastructure integrates genome assemblies, annotations, genetic maps and SNPs Also integrates gene/protein expression data, metabolic profiles and associated metadata Supports cross-omics analyses and data reuse	Cannabis-specific indirect/enabling evidence [148]	Role in biological control remains enabling rather than direct Sufficiently large harmonised BCA datasets are not yet available for robust predictive modelling
Step 8. Prospective multi-omics-informed SynCom design and experimental evaluation			
Microbiome profiling Hypothesis-generation network analysis Comparative genomics Strain-specific tracking Metatranscriptomics Metabolomics Community-reconstitution experiments	Cannabis microbiome datasets provide candidate taxa and contextual information Cannabis inoculant studies further indicate that microbial consortia can alter phytocannabinoid profiles, supporting concurrent crop-chemistry assessment during prospective SynCom testing Other plant systems provide proof-of-concept for defined community assembly and simplification Cross-pathosystem studies also provide methodological support for priority-effect testing, analysis of potentially influential community members, functional tracking and community reconstitution	Cannabis-specific indirect evidence [101,102,114,142] Cross-pathosystem support [116,127,149,150,152,153,154,155,156] Conceptual proposal	No disease-suppressive SynCom has yet been demonstrated for the cannabis pathosystems considered SynCom-design recommendations remain methodological extrapolations pending validation in cannabis disease systems Strain compatibility, priority effects, ecological stability and reproducibility across host genotypes and cultivation environments remain to be established directly in cannabis Potential non-target effects on resident beneficial taxa, community functional redundancy and microbial metabolic activity remain largely unexplored, particularly after repeated applications
Step 9. Formulation and application-oriented validation			
Formulation Microbial viability assessment Storage Delivery Strain/community monitoring	General BCA literature provides principles for formulation and maintenance of microbial viability Controlled cannabis studies provide initial efficacy evidence relevant to the subsequent evaluation of candidate BCAs under application-oriented conditions	Cannabis-specific direct evidence -controlled efficacy assays providing a basis for subsequent application-oriented assessment: propagation material [20], indoor whole-plant testing [44] and greenhouse whole-plant testing [52]; no direct formulation or production-scale validation Cross-pathosystem support [17,157] Conceptual proposal	Cannabis-specific information remains limited for shelf life and storage stability Compatibility with fertigation and other crop-protection practices and repeated application remain insufficiently characterised Reproducible efficacy under application-relevant cultivation conditions remains limited Ecological restructuring or loss of disease-suppressive function as experimental complexity increases remains largely unexplored
Integrated application-oriented performance assessment Integrated crop-quality assessment	The proposed framework integrates disease suppression with BCA/SynCom persistence Includes resident microbiome responses and crop performance Integrates cannabinoid and broader crop-chemistry profiles, microbiological quality at harvest and safety-associated traits as criteria for later-stage decisions; failure to meet predefined crop-chemistry or harvest-stage microbial-purity requirements should prevent progression towards production-scale validation, irrespective of disease-suppression efficacy. Includes preliminary comparative cost-effectiveness relative to existing disease-management practices as a later-stage advancement consideration.	Cannabis-specific indirect evidence [14,142,158] Cross-pathosystem support [157] Conceptual proposal	These endpoints have not yet been jointly integrated and experimentally evaluated within a cannabis-specific BCA-development framework under application-relevant conditions Full regulatory, manufacturing and economic assessment is a subsequent stage of commercial translation beyond the scope of the present framework Cannabis-specific comparative cost-effectiveness and techno-economic analyses of BCAs and SynComs are currently lacking.

## Data Availability

No new data were created or analysed in this study. Data sharing is not applicable to this article.

## Supplementary Materials

The following supporting information can be downloaded at: https://www.mdpi.com/article/10.3390/ijms27188024/s1.

## Abbreviations

The following abbreviations are used in this manuscript:

16S rRNA	16S ribosomal RNA
ABA	Abscisic acid
ACC	1-Aminocyclopropane-1-carboxylate
API	Application programming interface
ASV	Amplicon sequence variant
BCA	Biological control agent
CBD	Cannabidiol
CBDV	Cannabidivarin
CBDVA	Cannabidivarinic acid
CBG	Cannabigerol
CBGA	Cannabigerolic acid
CDPK	Calcium-dependent protein kinase
dPCR	Digital polymerase chain reaction
ERF1	Ethylene response factor 1
ET	Ethylene
GWAS	Genome-wide association study
HEL	Hevein-like protein
ISR	Induced systemic resistance
ITS	Internal transcribed spacer
JA	Jasmonic acid
LC-MS/MS	Liquid chromatography-tandem mass spectrometry
MAG	Metagenome-assembled genome
MAPK	Mitogen-activated protein kinase
MLO	Mildew Locus O
NB-ARC	Nucleotide-binding adaptor shared by APAF-1, R proteins and CED-4
NBS-LRR	Nucleotide-binding site-leucine-rich repeat
NHP	N-Hydroxypipecolic acid
NLR	Nucleotide-binding leucine-rich repeat receptor
NPR1	NONEXPRESSOR OF PATHOGENESIS-RELATED GENES 1
PAL	Phenylalanine ammonia-lyase
PAMP	Pathogen-associated molecular pattern
PCR	Polymerase chain reaction
PGPM	Plant growth-promoting microorganism
PGPR	Plant growth-promoting rhizobacteria
PM	Powdery mildew
PR1	Pathogenesis-related protein 1
PR2	Pathogenesis-related protein
PRR	Pattern-recognition receptor
qPCR	Quantitative polymerase chain reaction
QTL	Quantitative trait locus
RAD-seq	Restriction site-associated DNA sequencing
RNA	Ribonucleic acid
RNA-seq	RNA sequencing
RNS	Reactive nitrogen species
ROS	Reactive oxygen species
RT-qPCR	Reverse transcription quantitative polymerase chain reaction
SA	Salicylic acid
SAR	Systemic acquired resistance
SNP	Single-nucleotide polymorphism
SynCom	Synthetic microbial community
THC	Δ9-Tetrahydrocannabinol
